# Genome-wide association study meta-analysis of chronic widespread pain: evidence for involvement of the 5p15.2 region

**DOI:** 10.1136/annrheumdis-2012-201742

**Published:** 2012-09-06

**Authors:** Marjolein J Peters, Linda Broer, Hanneke L D M Willemen, Gudny Eiriksdottir, Lynne J Hocking, Kate L Holliday, Michael A Horan, Ingrid Meulenbelt, Tuhina Neogi, Maria Popham, Carsten O Schmidt, Anushka Soni, Ana M Valdes, Najaf Amin, Elaine M Dennison, Niels Eijkelkamp, Tamara B Harris, Deborah J Hart, Albert Hofman, Frank J P M Huygen, Karen A Jameson, Gareth T Jones, Lenore J Launer, Hanneke J M Kerkhof, Marjolein de Kruijf, John McBeth, Margreet Kloppenburg, William E Ollier, Ben Oostra, Antony Payton, Fernando Rivadeneira, Blair H Smith, Albert V Smith, Lisette Stolk, Alexander Teumer, Wendy Thomson, André G Uitterlinden, Ke Wang, Sophie H van Wingerden, Nigel K Arden, Cyrus Cooper, David Felson, Vilmundur Gudnason, Gary J Macfarlane, Neil Pendleton, P Eline Slagboom, Tim D Spector, Henry Völzke, Annemieke Kavelaars, Cornelia M van Duijn, Frances M K Williams, Joyce B J van Meurs

**Affiliations:** 1 Department of Internal Medicine, Erasmus Medical Center Rotterdam, Rotterdam, The Netherlands; 2 The Netherlands Genomics Initiative-sponsored Netherlands Consortium for Healthy Aging (NGI-NCHA), Leiden/Rotterdam, The Netherlands; 3 Department of Epidemiology, Erasmus Medical Center Rotterdam, Rotterdam, The Netherlands; 4 Laboratory of Neuroimmunology and Developmental Origins of Disease, University Medical Center Utrecht, The Netherlands; 5 Icelandic Heart Association Research Institute, Kopavogur, Iceland; 6 Aberdeen Pain Research Collaboration (Musculoskeletal Research), University of Aberdeen, Aberdeen, UK; 7 Arthritis Research UK Epidemiology Unit, Manchester Academic Health Science Centre, University of Manchester, Manchester, UK; 8 Mental Health and Neurodegeneration Group, School Community Based Medicine, University of Manchester, Manchester, UK; 9 Department of Medical Statistics and Bioinformatics, Section of Molecular Epidemiology, Leiden University Medical Centre, Leiden, The Netherlands; 10 Clinical Epidemiology Unit, Boston University School of Medicine, Boston, Massachusetts, USA; 11 Department of Twin Research and Genetic Epidemiology, King's College London, London, UK; 12 Institute for Community Medicine, University of Greifswald, Greifswald, Germany; 13 NIHR Musculoskeletal Biomedical Research Unit, University of Oxford, Oxford, UK; 14 MRC Lifecourse Epidemiology Unit, University of Southampton, Southampton General Hospital, Southampton, UK; 15 School of Biological Sciences, Victoria University of Wellington, Wellington, New Zealand; 16 Molecular Nociception Group, University College London, London, UK; 17 Intramural Research Program, Laboratory of Epidemiology, Demography, and Biometry, National Institute on Aging, Bethesda, Maryland, USA; 18 Department of Anaesthesiology, Erasmus Medical Center Rotterdam, Rotterdam, The Netherlands; 19 Aberdeen Pain Research Collaboration (Epidemiology Group), University of Aberdeen, Aberdeen, UK; 20 Department of Rheumatology, Leiden University Medical Center, Leiden, The Netherlands; 21 Department of Clinical Epidemiology, Leiden University Medical Center, Leiden, The Netherlands; 22 Centre for Integrated Genomic Medical Research, University of Manchester, Manchester, UK; 23 Department of Clinical Genetics, Erasmus Medical Center Rotterdam, Rotterdam, The Netherlands; 24 Medical Research Institute, University of Dundee, Dundee, UK; 25 Department of Medicine, University of Iceland, Reykjavik, Iceland; 26 Institute of Functional Genomics, Ernst Moritz Arndt University Greifswald, University of Greifswald, Greifswald, Germany; 27 NIHR Biomedical Research Unit, Nuffield Department of Orthopaedics, Rheumatology and Musculoskeletal Sciences, University of Oxford, Oxford, UK

**Keywords:** Gene Polymorphism, Fibromyalgis/Pain Syndromes, Epidemiology

## Abstract

**Background and objectives:**

Chronic widespread pain (CWP) is a common disorder affecting ∼10% of the general population and has an estimated heritability of 48–52%. In the first large-scale genome-wide association study (GWAS) meta-analysis, we aimed to identify common genetic variants associated with CWP.

**Methods:**

We conducted a GWAS meta-analysis in 1308 female CWP cases and 5791 controls of European descent, and replicated the effects of the genetic variants with suggestive evidence for association in 1480 CWP cases and 7989 controls. Subsequently, we studied gene expression levels of the nearest genes in two chronic inflammatory pain mouse models, and examined 92 genetic variants previously described associated with pain.

**Results:**

The minor C-allele of rs13361160 on chromosome 5p15.2, located upstream of chaperonin-containing-TCP1-complex-5 gene (CCT5) and downstream of FAM173B, was found to be associated with a 30% higher risk of CWP (minor allele frequency=43%; OR=1.30, 95% CI 1.19 to 1.42, p=1.2×10^−8^). Combined with the replication, we observed a slightly attenuated OR of 1.17 (95% CI 1.10 to 1.24, p=4.7×10^−7^) with moderate heterogeneity (I2=28.4%). However, in a sensitivity analysis that only allowed studies with joint-specific pain, the combined association was genome-wide significant (OR=1.23, 95% CI 1.14 to 1.32, p=3.4×10^−8^, I2=0%). Expression levels of Cct5 and Fam173b in mice with inflammatory pain were higher in the lumbar spinal cord, not in the lumbar dorsal root ganglions, compared to mice without pain. None of the 92 genetic variants previously described were significantly associated with pain (p>7.7×10^−4^).

**Conclusions:**

We identified a common genetic variant on chromosome 5p15.2 associated with joint-specific CWP in humans. This work suggests that CCT5 and FAM173B are promising targets in the regulation of pain.

## Introduction

Chronic widespread pain (CWP) is a common disorder, affecting about 10% of the general population.^1^ The prevalence of CWP increases with age for both men and women, but is more common in women at any age.^1^ CWP represents a major underestimated health problem and is associated with substantial impairment and a reduced quality of life. It has been related to a number of physical and affective symptoms such as fatigue, psychological distress and somatic symptoms.^1^ ^2^ Chronic musculoskeletal pain is one of the most common conditions seen in rheumatology clinics and accounts for 6.2% of the total healthcare costs in The Netherlands every year.^3^ Further research is needed to be able to understand the causal mechanisms and optimal treatment for CWP patients.

CWP causally relates to an initial local pain stimulus, such as an acute injury or athletic injuries, or another pain state such as low back pain or local pain due to osteoarthritis (OA) or rheumatic arthritis (RA).^4–6^ However, most injured subjects do not develop CWP, and only a proportion of patients with OA or RA develop CWP. We therefore hypothesise that several discrete stimuli may initiate CWP via a common final pathway that involves the generation of a central pain state through the sensitisation of second order spinal neurons.

CWP is a complex trait since both environmental and genetic factors play a role in the aetiology. Heritability estimates of twin studies suggest that 48–52% of the variance in CWP occurrence is due to genetic factors, implying a strong genetic component.^7^ A number of studies have examined genetic variants for CWP. These candidate gene studies examined polymorphisms in genes involved in both the peripheral and the central nervous system.^8^ In particular, genes involved in neurotransmission (pathway of dopamine and serotonin^9–19^), and genes important for the hypothalamic–pituitary–adrenal axis have been considered.^20^ A number of genetic variants in these candidate genes were found to be associated with CWP, individual pain sites or experimental pain. However, no consistent significant associations have been demonstrated.

The most studied gene in relation to pain is catechol-O-methyltransferase (COMT), an enzyme that degrades neurotransmitters including dopamine. The variant allele of rs4680 (or V158M) results in reduced enzymatic activity due to its effect on thermostability,^21^ and has been associated with reduced opioid activity in response to painful stimuli resulting in increased pain sensitivity.^22^ But also for COMT, no consistent results have been observed in genetic association studies.^13^ ^23–29^

Overall, the results have been conflicting, which is likely due to the modest sample sizes used and paucity of replication. In general, candidate studies are biased by previous knowledge of the aetiology of the disease under study. Since knowledge about the pathophysiology of CWP is poor, the chances of success using this approach are low. Therefore our objective was to identify genetic variants involved in CWP by means of a large-scale hypothesis-free genome-wide association study (GWAS) meta-analysis including 2788 cases and 13 780 controls. To our knowledge, this is the first study presenting a large-scale GWAS meta-analysis of chronic pain. The prevalence of CWP is approximately two times higher in women than in men and there is strong evidence that women tolerate less thermal and pressure pain than men.^30^ Therefore only women were included in this study to reduce heterogeneity and thereby increase power.

## Materials and methods

**Figure 1:**
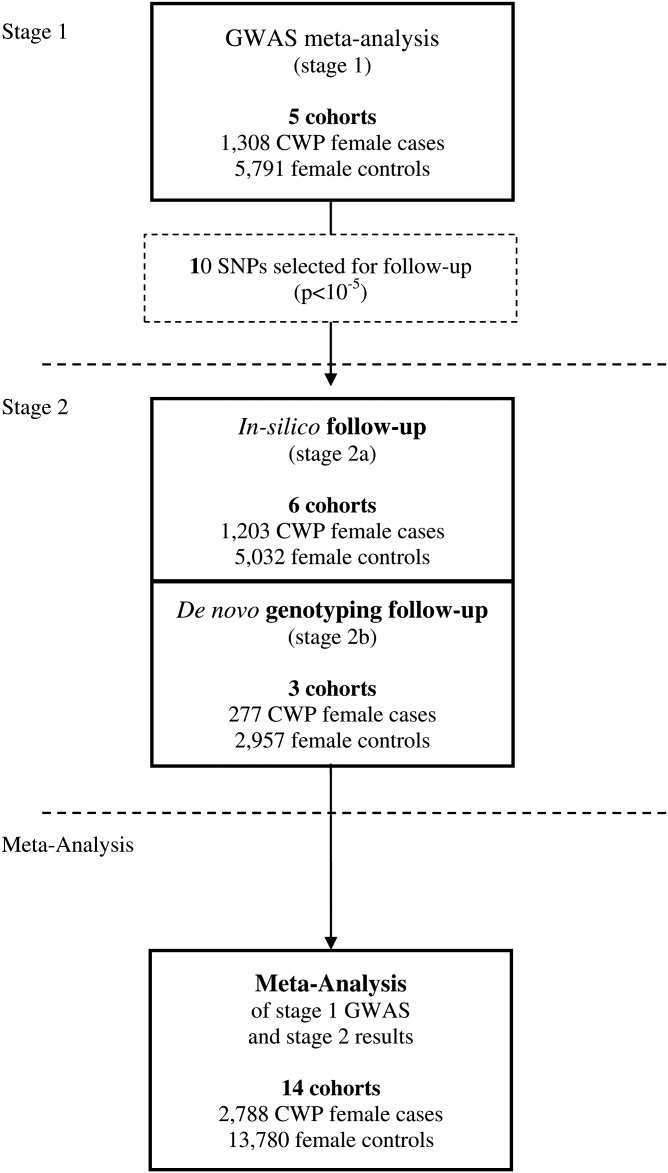
Study outline. CWP, chronic widespread pain; GWAS, genome-wide association study.

We performed a meta-analysis (stage 1) of GWAS data of 1308 female Caucasian CWP cases and 5791 female Caucasian controls, derived from five studies, and focused our follow-up efforts on the single-nucleotide polymorphisms (SNPs) with suggestive evidence of association (p<1×10^−5^) with CWP (stage 2). The study outline is summarised in figure 1.

### Phenotype

CWP was defined as subjects having pain in the left side of the body, in the right side of the body, above the waist, below the waist, and in the axial skeleton (following the Fibromyalgia Criteria of the American College of Rheumatology^2^). Controls were defined as subjects not having CWP. Subjects using analgesics (ATC code: N02^31^) were excluded from the control group. Detailed descriptions of the study specific inclusion criteria are presented in supplementary table S1.

### Study design summary

We combined the summary statistics of GWAS in a meta-analysis comprising 1308 CWP female Caucasian cases and 5791 female Caucasian controls (stage 1). We focused our follow-up efforts on the SNPs with suggestive evidence of association (p<1×10^−5^) with CWP in 1480 CWP cases and 7989 controls available for replication (stage 2).

#### Subjects

A full detailed description of all study cohorts is presented in table 1 and in the supplementary methods section. For the stage 1 analysis, we included studies from The Netherlands (the Erasmus Rucphen Family study (ERF study),^32^ Rotterdam Study I, II and III (RS-I, RS-II and RS-III)^33^), and the UK (TwinsUK^34^ ^35^). All studies were approved by their institutional ethics review committees and all participants provided written informed consent. For our stage 2 analysis, we sought follow-up samples with pre-existing GWAS in silico data (stage 2a) as well as de novo genotyping (stage 2b). The studies are from the UK (the British 1958 Birth Cohort (1958BC),^23^ ^36–38^ the Chingford Study (CHINGFORD),^39^ ^40^ the Dyne Steel DNA Bank for Aging and Cognition (DSDBAC),^41^ the EPIdemiological study of FUNctional Disorders (EPIFUND),^20^ and the Hertfordshire Cohort Study (HCS)^42^); from Iceland (the Age, Gene/Environment Susceptibility Study (AGES)^43^); from the USA (the Framingham Osteoarthritis Study (FOA)^44^); from The Netherlands (the Genetics osteoARthritis and Progression Study (GARP)^45^); and from Germany (the Study of Health In Pomerania (SHIP)^46^ ^47^). All studies were approved by the local ethics committees and all participants provided written informed consent.

**Table 1:** Overview of all participating studies

Study (stage)	Reference article	Study design	Ethnic origin	Country of origin	Medication	Age/BMI	Mean age (y)	No. of CWP cases	No. of CWP controls
Stage 1									
ERF study	32	Family based cohort	Caucasian	The Netherlands	Y	Y	46.4	149	665
RS-I	33	Population based cohort	Caucasian	The Netherlands	Y	Y	69.4	563	1892
RS-II	33	Population based cohort	Caucasian	The Netherlands	Y	Y	67.9	110	668
RS-III	33	Population based cohort	Caucasian	The Netherlands	Y	Y	56.3	85	868
TwinsUK	34 35	Twins based cohort	Caucasian	UK	Y	Y	51.9	401	1698
Total no. of samples							59.7	1308	5791
Stage 2a									
1958BC	23 36–38	Prospective birth cohort	Caucasian	UK	N	Y (born in 1958)	NA	315	2206
AGES	43	Population based cohort	Caucasian	Iceland	Y	Y	76.5	173	1204
DSDBAC	41	Population based cohort	Caucasian	UK	Y	Age only	80.1	81	219
FOA	44	Population based cohort	Caucasian	USA	Y	Y	59.3	384	814
GARP	45	Case control based	Caucasian	The Netherlands	Y	Y	58.5	67	925*
SHIP	46 47	Population based cohort	Caucasian	Germany	Y	Y	57.6	183	589
Stage 2b									
CHINGFORD	39 40	Population based cohort	Caucasian	UK	Y	Y	56.6	48	337
EPIFUND	20	Population based cohort	Caucasian	UK	N	Age only	49.0	139	503
HCS	42	Population based cohort	Caucasian	UK	Y	Y	66.4	90	2117
Total no. of samples								1480	7989

*GARP consists of clinical and radiographically confirmed osteoarthritis case only; therefore we used 925 randomly chosen Rotterdam Study samples as controls.

Age/BMI Y, age and BMI data are available; Age only, no BMI data are available; AGES, Age, Gene/Environment Susceptibility study Reykjavik; BMI, body mass index; CWP, chronic widespread pain; CHINGFORD, Chingford 1000 Women Study; DSDBAC, Dyne Steel DNA Bank for Ageing and Cognition; ERF study, Erasmus Rucpen Family study; EPIFUND, EPIdemiological study of FUNctional Disorders study; HCS, Hertfordshire Cohort Study; FOA, Framingham Osteoarthritis Study; GARP, Genetics OsteoArthritis and Progression study Leiden; Medication Y, information about medication use available; Medication N, medication use not available; RS, Rotterdam Study; SHIP, Study of Health In Pomerania; TwinsUK, the UK Adult Twin Registry; 1958BC, 1958 Birth Cohort.

#### Genotyping, quality control and imputation

Genotyping of the stage 1 cohorts was done by Illumina Infinium HumanHap550 Beadchip (RS-I and RS-II), the Illumina Infinium HumanHap610 (RS-II, RS-III, and TwinsUK), or the Illumina Infinium HumanHap300 (ERF and TwinsUK). More details about the genotyping, quality control (QC), and imputation are shown in the supplementary methods section. Complete information on genotyping protocols and QC measures for all stage 1 cohorts is described in the supplementary material (see supplementary table S2). Detailed descriptions of the QC and imputation procedures are provided in the supplementary material (see supplementary table S3).

Genotypes of the stage 2a studies (1958BC, AGES, DSDBAC, FOA, GARP and SHIP) were obtained from SNP arrays and imputed data. Where unavailable, proxy SNPs were selected based on high linkage disequilibrium (LD). The stage 2b studies (CHINGFORD, EPIFUND and HCS) performed de novo genotyping, using both Sequenom iPLEX and TaqMan-based assays (supplementary methods). Genotyping platforms, calling algorithms, quality control before imputation, imputation methods and analysis software used were all study-specific (see supplementary tables S4 and S5). The explicit number of follow-up SNPs genotyped in the different studies and whether the original or a proxy SNP was used is summarised in supplementary table S6.

#### GWAS analysis in the stage 1 studies

CWP was analysed as a binary trait (cases vs controls) using logistic regression under an additive model with adjustment for age and body mass index (see supplementary table S7). To adjust for population substructure, we included the four most important PCs as covariates in the regression analysis of RS-I, RS-II and RS-III. These PCs were derived from a multidimensional scaling analysis of identity-by-state distances, using PLINK software.^48^ Detailed descriptions of the GWAS methods are provided in supplementary table S8).

#### Stage 1: GWAS meta-analysis

p Values for association were combined using the Meta-Analysis Tool for genome-wide association scans (METAL).^49^ The genomic control method^50^ as implemented in METAL was used to correct for any residual population stratification or relatedness not accounted for by the four most important PCs. A p value <5×10^−8^ was considered genome-wide significant while a p value <1×10^−5^ was considered suggestive.^51^ Power calculations were performed using CaTS software (http://www.sph.umich.edu/csg/abecasis/CaTS/). Using Bonferroni correction (p<5×10^−8^), power calculations showed that we had approximately 80% power to detect an OR of 1.30 for SNPs with a minor allele frequency (MAF) of 0.43, given a disease prevalence of 10% for 1308 cases and 5791 controls in the discovery group. Using a p value <1×10^−5^, we had 80% power to detect an OR of 1.25.

#### SNP selection for replication

We aimed to select SNPs for replication (stage 2) that were enriched for signals of association with CWP. All SNPs with suggestive evidence for association in the stage 1 analyses were selected and separated into independent loci by taking the most significantly associated SNP and eliminating all SNPs that have a HapMap CEU pairwise correlation coefficient r^2^>0.8 with that SNP using the PLINK software.

#### Meta-analysis of stage 1 and stage 2 results

We combined the stage 1 and stage 2 association results to derive a combined meta-analysis for the suggestively associated loci. METAL was used to conduct a fixed-effects meta-analysis as in stage 1. Estimated heterogeneity variance and forest plots were generated using comprehensive meta-analysis (http://www.meta-analysis.com).

#### Functional analysis of associated SNPs

To determine whether the associated SNPs have any regulatory effect on gene expression levels, we checked their effect (and the effect of the linked SNPs) on the expression levels of their neighbouring genes. We used the 1000 genomes data in the SNAP software^52^ ^53^ to identify those SNPs having LD thresholds of r^2^>0.1. We searched two publicly available eQTL databases: the NCBI GTEx (Genotype-Tissue Expression) eQTL browser (http://www.ncbi.nlm.nih.gov/gtex/GTEX2/gtex.cgi) and the expression Quantitative Trait Loci database (http://eqtl.uchicago.edu/cgi-bin/gbrowse/eqtl/). We used SIFT^54^ to predict whether the coding non-synonymous variant causing an amino acid substitution affects protein function.

### RNA expression analyses in mice

For functional follow-up, two independent mouse models of inflammatory pain were studied. The first model was based on carrageenan injections; female C57Bl/6 mice received an intraplantar injection of 20 μl λ-carrageenan (2% (w/v), Sigma Aldrich, Zwijndrecht, the Netherlands) in saline in both hind paws.^55^ The second model was based on Complete Freund's Adjuvant (CFA) injections; male C57Bl/6 mice (Harlan Laboratories) received an intraplantar injection of 20 μl CFA (Sigma-Aldrich) in saline in both hind paws.^56^ Controls were injected with saline only. At day 3 (after CFA injection) or day 6 (after carrageenan injection), thermal sensitivity (heat withdrawal latency time) was measured using the Hargreaves (IITC Life Science, Woodland Hills, California, USA) test as described.^57^ Intensity of the light beam was chosen to induce heat withdrawal latency time of approximately 8 s at baseline.

After measurement the mice were sacrificed and the lumbar (L2–L5) spinal cord and the dorsal root ganglions (DRG) (L2–L5) were isolated. These areas of spinal cord and DRG were selected because pain transmission from the hind paws is mediated via primary sensory neurons that have their cell bodies in the lumbar DRG, and transmit the signal to the lumbar spinal cord through sensory fibres in the dorsal roots. Total RNA was isolated and mRNA levels of Cct5 and Fam173b were measured in the spinal cord and the DRG. For more details, see the supplementary methods section.

All experiments were performed in accordance with international guidelines and approved by the experimental animal committee of the University Medical Center Utrecht (carrageenan experiment) or the UK Home Office Animals (Scientific Procedures) Act 1986 (CFA experiment). Mice used for the carrageenan experiment were bred and maintained in the animal facility of the University of Utrecht (The Netherlands).

### Systemic review of genetic variants previously described

We systematically searched for associations earlier reported with pain in the HugeNavigator PhenoPedia database.^58^ We used the search term ‘pain’ and checked all publications for genes and SNPs associated with pain at least twice. Genes and SNPs associated with drug therapy, facial pain, migraine and postoperative pain were excluded. For all reported SNPs, we examined their association with CWP in our stage 1 meta-analysis. The significance threshold was set at p<8×10^−4^ using Bonferroni correction for 65 independent genetic loci. Again, power calculations were performed using CaTS software (http://www.sph.umich.edu/csg/abecasis/CaTS/). With an α level of 8×10^−4^, power calculations showed that we had approximately 80% power to detect an OR of 1.22 for SNPs with a minor allele frequency of 20% or higher.

## Results

### GWAS meta-analysis for CWP

**Figure 2:**
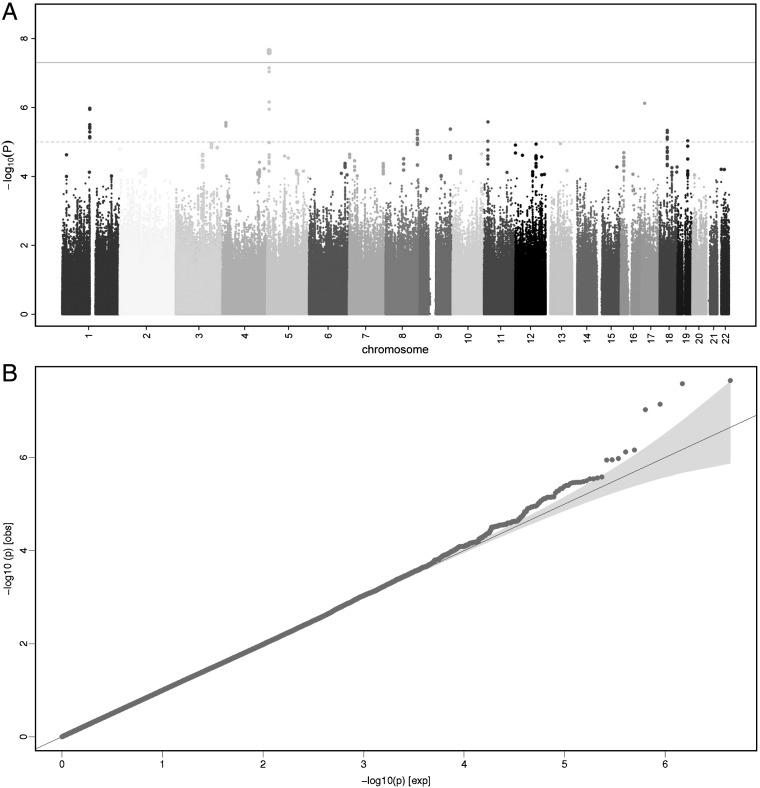
Genome-wide association results for chronic widespread pain (CWP) (stage 1). (A) Manhattan plot showing the p value of association tests for about 2 million SNPs with CWP in the stage 1 meta-analysis. SNPs are plotted on the x-axis according to their position on each chromosome. On the y-axis, the association p values with CWP are shown (as −log 10 p values). The grey solid horizontal line represents the p value threshold of 5×10^−8^ (genome-wide significance). The grey dashed horizontal line represents the p value threshold of 1×10^−5^ (the level for suggestive evidence): SNPs in loci reaching 1×10^−5^ were tested for replication. (B) Quantile–quantile (QQ) plot of SNPs. The blue area represents the 95% CI around the test statistics. A QQ plot compares the additive model statistics to those expected under the null distribution using fixed effects for all analysed HapMAP CEU imputed SNPs passing quality control criteria. This figure is only reproduced in colour in the online version.

The Manhattan plot and quantile–quantile plot of the initial stage 1 meta-analysis are presented in figure 2. In total, 2 224 068 SNPs (directly genotyped or imputed) were tested for association. The overall genomic control lambda (λ_GC_) was 1.007, indicating no significant population stratification. We identified two SNPs which were genome-wide significant (p<5×10^−8^), and another 39 SNPs with suggestive evidence for association (p<1×10^−5^) located in 10 independent genomic regions. The most significant association was observed for two imputed highly correlated SNPs (r^2^=0.97) located upstream of the chaperonin-containing-TCP1-complex-5 gene (CCT5) and downstream of the FAMily with sequence similarity 173, member B gene (FAM173B) (rs13361160, p=1.2×10^−8^ and rs2386592, p=2.6×10^−8^). For both SNPs, the minor allele (MAF=43%) was associated with a 30% higher risk for CWP (OR=1.30, 95% CI 1.19 to 1.42).

### Meta-analysis of GWAS replication

**Figure 3:**
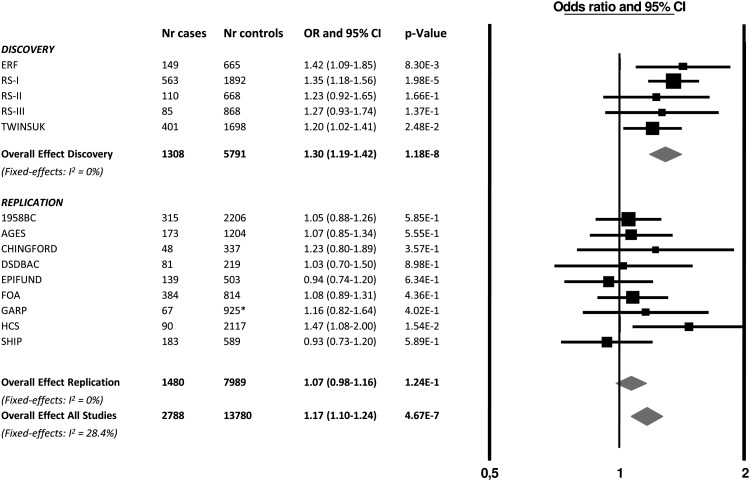
Forest plot of the association of rs13361160 SNP with chronic widespread pain (CWP). Study specific estimates and summary association between rs13361160 and CWP are shown. This figure is only reproduced in colour in the online version.

For the 10 independent SNPs with suggestive evidence, we pursued in silico replication data in six studies (stage 2a: 1203 CWP cases and 5032 controls) and performed de novo genotyping in subjects from three additional studies (stage 2b: 277 CWP cases and 2957 controls) (a detailed description of the studies is presented in table 1 and supplementary methods). The summary results of the stage 1 and 2 meta-analysis are presented in table 2. After combining the results of stage 1 and stage 2, the top SNP was rs13361160 (OR=1.17, 95% CI 1.10 to 1.24, p=4.7×10^−7^, I^2^=28.4%). Figure 3 shows a forest plot of the association of rs13361160 with CWP across the stage 1 and stage 2 studies. The overall effect in the replication studies (stage 2 studies) was in a consistent direction but not significant (OR=1.06, 95% CI 0.98 to 1.16, p=0.16). In the combined analysis, moderate heterogeneity was observed (I^2^=28.4%). supplementary table S1 shows the different pain assessment methods used in the different studies to define CWP. Since four out of five stage 1 studies included joint-specific pain only (ERF, RS-I, RS-II and RS-III), we performed a sensitivity analysis in which stage 2 cohorts using non-joint pain were excluded (1958BC, DSDBAC, EPIFUND, HCS and SHIP). This resulted in a combined OR of 1.23 (95% CI 1.14 to 1.32, p=3.4×10^−8^, I^2 ^= 0%). An overview of the results of the combined meta-analysis and the separate stage 1 and stage 2 analyses is presented in table 3.

**Table 2:** Association results of the 10 top hits

SNP information					Gene information		Stage 1			Stage 2			Stage 1 and 2 (combined)		
SNP ID	CHR	Minor allele (MA)	Other allele (OA)	MAF (%)	Nearest gene	Distance to gene (kb)	OR (minor allele)	95% CI	p Value	OR (minor allele)	95% CI	p Value	OR (minor allele)	95% CI	p Value
rs13361160	5	c	t	43.5	FAM173B	56.7	1.30	1.18 to 1.42	1.18×10^−8^	1.06	0.98 to 1.16	0.161	1.17	1.10 to 1.24	4.67×10^−7^
rs8065610	17	a	c	38.7	PMP22	41.9	1.26	1.15 to 1.38	3.86×10^−7^	0.93	0.85 to 1.02	0.119	1.08	1.02 to 1.16	1.20×10^−2^
rs12132674	1	a	g	29.5	HMGCS2	0.0	1.28	1.16 to 1.41	9.29×10^−7^	1.07	0.97 to 1.17	0.165	1.16	1.09 to 1.24	1.23×10^−5^
rs11606304	11	g	t	9.07	TPH1	0.0	0.55	0.43 to 0.70	1.47×10^−6^	0.99	0.81 to 1.22	0.934	0.78	0.66 to 0.91	1.64×10^−3^
rs7680363	4	a	t	6.42	PROM1	0.0	1.52	1.28 to 1.80	1.72×10^−6^	1.04	0.86 to 1.25	0.688	1.27	1.12 to 1.44	1.52×10^−4^
rs4837492	9	c	t	4.51	FREQ	56.2	1.23	1.13 to 1.34	2.96×10^−6^	0.97	0.89 to 1.07	0.568	1.10	1.03 to 1.17	2.68×10^−3^
rs524513	18	t	c	18.2	BRUNOL4	0.0	1.29	1.16 to 1.44	4.00×10^−6^	0.96	0.85 to 1.09	0.537	1.13	1.04 to 1.23	2.75×10^−3^
rs7835968	8	g	a	12.7	KHDRBS3	175.8	1.34	1.18 to 1.52	4.26×10^−6^	0.98	0.86 to 1.12	0.787	1.16	1.06 to 1.27	1.43×10^−3^
rs2249104	11	t	c	8.8	MYOD1	3.7	1.42	1.22 to 1.64	4.57×10^−6^	0.99	0.84 to 1.17	0.882	1.21	1.08 to 1.35	8.78×10^−4^
rs17796312	19	g	a	33.1	FBL	7.1	1.24	1.13 to 1.37	9.79×10^−6^	1.08	0.97 to 1.2	0.166	1.17	1.09 to 1.25	2.56×10^−5^

CHR, chromosome; MA, minor allele or effect allele (minor allele=effect allele); MAF, minor allele frequency (%);OA, other allele.

**Table 3:** Top hit association results

Type of analysis		Stage 1		Stage 2		Stage 1 and 2 (combined)	
SNP tested	Adjustments	OR (95% CI)	p Value	OR (95% CI)	p Value	OR (95% CI)	p Value
rs13361160	Age, BMI, and 4 PCs	1.30 (1.19 to 1.42)	1.18×10^−8^	1.06 (0.98 to 1.16)	0.16	1.17 (1.10 to 1.24)	4.67×10^−7^
(minor allele=C, other allele=T, MAF=43.5%)							
Sensitivity analysis; joint pain only							
rs13361160	Age, BMI, and 4 PCs	1.30 (1.19 to 1.42)	1.18×10^−8^	1.10 (0.97 to 1.25)	0.15	1.23 (1.14 to 1.32)	3.43×10^−8^
(minor allele=C, other allele=T, MAF=43.5%)							

In both analyses the effect estimates of the models refer to the minor allele (=effect allele).

BMI, body mass index; MAF, minor allele frequency.

### Functional analysis of rs13361160 and rs2386592

**Figure 4:**
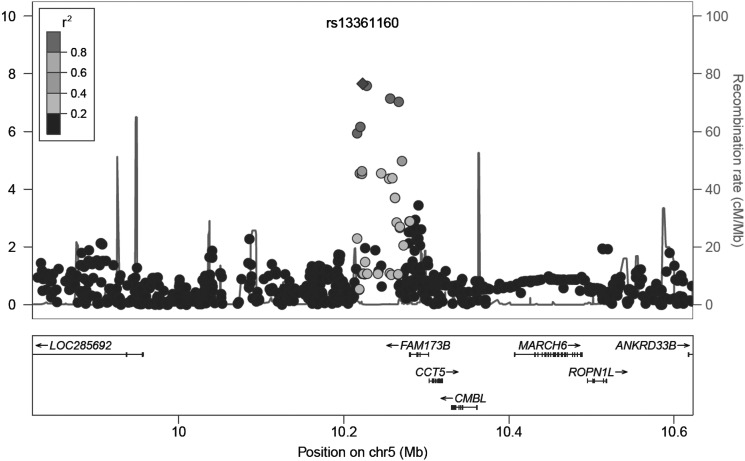
Regional plot of locus 5p15.2. On the x-axis, SNPs are plotted according to their position in a 400-kb window around rs13361160. On the y-axis, the association p values with chronic widespread pain are shown (as −log 10 p values). The purple diamond highlights the most significant SNP rs13361160. Blue peaks indicate recombination sites, and the SNPs surrounding the most significant SNP are colour coded to identify their strength of linkage disequilibrium with the most significant SNP (pairwise r^2^ values of the HapMap CEU samples). Genes and the direction of transcription are shown at the bottom of the plot. This figure is only reproduced in colour in the online version.

The SNPs rs13361160 and rs2386592 (r^2^=0.97) are annotated to the 5p15.2-region and located 81 kb upstream of CCT5 and 57 kb downstream of FAM173B (figure 4). We tested whether rs13361160 and rs2386592 and their linked SNPs (r^2^>0.1) affected gene expression levels of CCT5 or FAM173B. In total, we identified 130 SNPs in LD with our top SNPs, of which two SNPs were located in the coding region: one synonymous SNP rs1042392 in the CCT5 gene (r^2^=0.16, D′=0.85) and one non-synonymous SNP rs2438652 in the FAM173B gene (r^2 ^= 0.17, D′=1.0) (see supplementary table S9). The minor allele of rs2438652 causes a threonine-to-methionine substitution (T75M) which is thought to be functionally neutral. SNPs rs13361160 and rs2386592 were not recorded as influencing the expression levels of CCT5 and FAM173B, however the linked intronic SNP rs2445871 (r^2^=0.14 for both) had a direct eQTL effect on FAM173B expression levels in liver tissue.^59^

### RNA expression analysis in mice

We studied gene expression levels of the two nearest genes, Cct5 and Fam173b, in the lumbar spinal cord and the DRG in two independent mouse models of chronic inflammatory pain. In both the carrageenan treated group and the CFA treated group, mice had shorter heat withdrawal latency times than mice injected with saline only, confirming enhanced pain sensitivity (p<0.001) (see supplementary figure S1).

**Figure 5:**
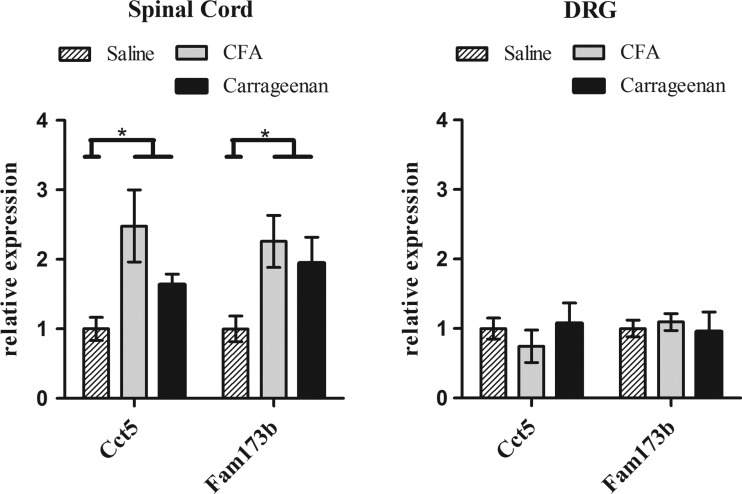
Quantitative PCR analysis of gene expression levels in the lumbar (L2–L5) spinal cord (A) and the dorsal root ganglions (DRG) (B) of mice after intraplantar saline (n=3), carrageenan (n=4), and Complete Freund's Adjuvant (CFA) (n=4) injection. Spinal cord and DRG were collected and analysed for RNA levels of Cct5 and Fam173b. Data were normalised for Gapdh and β-actin (housekeeping genes) expression. Data are expressed as mean±SEM, *=p<0.05.

The results from the multivariate analysis using the two genes (Cct5 and Fam173b examined as dependent variables), the different treatments (saline, carrageenan and CFA) and the different tissues (DRG and spinal cord) confirmed that there is a significant treatment effect for Cct5 (F(2,25)=3.399, p=0.0049), as well as for Fam173b (F(2,25)=4.911, p=0.016). Moreover, both genes showed a significant tissue effect (Cct5: F(1,25)=13.595, p=0.001, and Fam173b: F(1,25)=13.522, p=0.001), as well as a significant interaction between tissue and treatment (Cct5: F(2,25)=6.424, p=0.006, and Fam173b: F(1,25)=4.196, p=0.027) (figure 5). These findings indicate that in spinal cord but not in DRG, both Fam173b and Cct5 expression levels were up-regulated in response to two different inducers of inflammatory pain. DRG Fam173b and Cct5 expression levels in CFA/carrageenan-treated mice were indistinguishable from saline-treated mice.

### Candidate SNPs previously associated with chronic pain

We examined whether genetic variants previously described for association with pain were associated with CWP in our large stage 1 meta-analysis. We identified a total of 44 genes, of which 136 SNPs had been reported at least twice with any pain phenotype (excluding facial pain, migraine, postoperative pain and response to drug therapy), and we examined the association of these 136 SNPs with CWP in the GWAS stage 1 meta-analysis. Out of 136 candidate SNPs, we were able to check 92 common SNPs (MAF>5%) in 65 independent genetic loci (see supplementary table S10). Five SNPs had a too low MAF (<=5%) and 39 SNPs were not genotyped or imputed in our meta-analysis. None of the earlier reported SNPs passed the significance threshold (p<8×10^−4^). Interestingly, the strongest associated SNPs are located in three genes that have been reported to be associated with pain phenotypes most frequently: COMT, GCH1 (GTP cyclo-hydrolase 1) and OPRM1 (mu opioid receptor). The effects of the SNPs in GCH1 are in the same direction as reported earlier^60–62^: individuals having the minor allele for rs10483639, rs4411417 or rs752688 have 15% less pain than those exhibiting the common alleles. The effect of the SNP rs599548 in OPRM1 is also in the same direction as reported earlier^63^: those having the minor allele for rs599548 have 19% more pain than those exhibiting the major allele. The two COMT SNPs are in weak LD with the well-known amino acid changing variant rs4860, but previously have not been reported to be significantly associated with pain.^23^ ^64^ We have found a protective effect for the minor allele of rs2020917 (those having a minor allele have 15% less pain) and an adverse effect for the minor allele of rs5993883: those having the minor allele of rs5993883 have 14% more pain.

## Discussion

In this study, we identified a genetic variant near CCT5 and FAM173B to be associated with CWP. Chronic pain coincided with higher RNA expression of Cct5 and Fam173b in the lumbar spinal cord of mouse models of inflammatory pain. This finding indicates that both genes in the 5p15.2 region are regulated in the context of inflammatory pain.

Interestingly, Bouhouche *et al*^65^ reported a human pedigree in which a CCT5 mutation caused hereditary sensory neuropathy (Online Mendelian Inheritance in Man (OMIM) ID=610150), a syndrome characterised by a sensory deficit in the distal portion of the lower extremities, chronic perforating ulcerations of the feet and progressive destruction of underlying bones. Symptoms can include pain and numbness, tingling in the hands, legs or feet, and extreme sensitivity to touch. CCT5 is a subunit of the chaperonin containing t-complex polypeptide 1 (TCP-1) which assists in protein folding and assembly in the brain.^66^ CCT5 interacts with the serine/threonine-protein phosphatase 4 catalytic subunit PP4C.^67–69^ Zhang *et al*^70^ confirmed that protein phosphates like PPP4C may have a regulatory effect on the central sensitisation of nociceptive transmission in the spinal cord. Interestingly, sensitisation is thought to contribute to chronic inflammatory pain.^71^ Since the function of the FAM173B gene is not yet known, it is difficult to postulate the mechanism by which this gene could influence CWP. Further research into the genes in this locus is needed to ascertain whether either or both CCT5 and FAM173B are driving the observed association.

By combining the effects across the different stage 2 studies, moderate heterogeneity was observed in the meta-analysis. This heterogeneity might be caused by different pain assessment methods used by the stage 2 cohorts. In particular, four cohorts asked the participants about joint pain specifically, while the other five also included non-joint pain. When the non-joint pain phenotype were excluded, the heterogeneity across the cohorts reduced to 0% and the overall p value for rs13361160 now reached genome-wide significance by combining the stage 1 and stage 2 effects. This might suggest that indeed phenotype heterogeneity was introduced by including non-joint pain. In general, it is anticipated that pain is a very complex trait, with different aetiological pathways introducing phenotypic heterogeneity.

A limitation of our study is that we were not able to examine possible phenotype subgroups, such as individuals with RA, a chronic systemic inflammatory disorder that principally affects the synovial joints. Stratifying these groups of individuals might serve to increase power to find genetic loci. We here decided to analyse all CWP cases together, based on the hypothesis that several discrete stimuli need to initiate CWP via a common final pathway that involves the generation of a central pain state through the sensitisation of second order spinal neurons. In addition, the prevalence of RA is very low (about 0.5–1%),^72^ and the earlier defined GWAS hits for RA (ie, the HLA locus)^73^ were not in our top list. So, we assume the results were not dominated by this small number of individuals with RA.

It would be helpful to dissect the phenotype of pain into quantitative sub-phenotypes, for example by measuring pain sensitivity and pain thresholds for temperature or pressure,^74^ or by examining functional MRIs.^75^ The use of quantitative and possibly more objective pain measurements in response to painful stimuli (rather than reported pain) will be of pivotal importance for future pain research. Because we have focused on the clinical pain definition using questionnaires and pain homunculus, we accept that we may have missed true pain susceptibility alleles. However, this study represents the largest genome-wide meta-analysis looking into the genetics of human CWP to date. The experiments in two independent mouse models of chronic inflammatory pain showed that the expression of Cct5 and Fam173b was higher in the lumbar spinal cord of mice with chronic inflammatory pain but not in DRG. In the spinal cord, the expression profiles of both genes were up-regulated in response to two different inducers of inflammatory pain. These findings indicate that both genes in the 5p15.2 region are co-regulated in the spinal cord during inflammation-induced pain in both independent pain models, thereby possibly contributing to the neurobiology of pain. In the lumbar DRG, containing the cell bodies of the primary sensory neurons that detect pain signals from the hind paws, Cct5 and Fam173b gene expression levels did not change by inflammation. Because of these complementary results from the two independent tissues (spinal cord and DRG), we hypothesise that the 5p15.2 region is likely to play a role in spinal central pain processing and not in regulating primary sensory neuron responses.

In the study of candidate genes previously reported to be associated with a pain phenotype, we showed that none of the 92 studied variants were significantly associated with CWP in our GWAS meta-analysis. This can be explained by the fact that many of the previous reported loci were studied in relative modest sample sizes and in a large variety of pain phenotypes.^76^ Power calculations show that we had approximately 80% power to detect an OR as low as 1.22 for SNPs with an allele frequency of 20% or higher. So, even in this large meta-analysis, power was still modest to detect small ORs and we therefore cannot exclude smaller effect sizes of the tested variants, resulting in lack of reproducibility.^77^ This lack of reproducibility of SNPs in candidate genes in large GWAS meta-analyses has been shown before for other phenotypes such as bone mineral density (BMD).^78^ It is interesting to note that among the candidate SNPs, the strongest associated ones were located in the three most studied pain genes, COMT, GCH1 and OPRM1. The directions of the effects of these SNPs were the same as reported earlier, which would support true associations.

In conclusion, our study reports a GWAS meta-analysis on CWP. We identified the genetic variant rs13361160 at the 5p15.2 locus, located 81 kb upstream of the CCT5 gene and 57 kb downstream of the FAM173B gene, to be associated with CWP. We showed an increase in expression levels of Cct5 and Fam173b in the spinal cord of inflammatory pain models of mice, and since these genes both seem to influence the central mechanism of sensitisation, they may represent a novel pathway involved in pain sensation.

## Supplementary Material

Web supplement
