# Supplementary material for: Genome-wide association study meta-analysis of chronic widespread pain: evidence for involvement of the 5p15.2 region
Source: Ann Rheum Dis. 2012 Sep 6;72(3):427–36. doi: 10.1136/annrheumdis-2012-201742 (PMC3691951; doi:10.1136/annrheumdis-2012-201742)
Supplement: Web supplement [file annrheumdis-2012-201742-s1.pdf]

# Supplementary: Genome-wide association study meta-analysis of chronic widespread pain: evidence for involvement of the 5p15.2 region

Marjolein J. Peters[1,2*], Linda Broer[3*], Hanneke L.D.M. Willemen[4*], Gudny Eiriksdottir[5], Lynne J. Hocking[6], Kate L. Holliday[7], Michael A. Horan[8], Ingrid Meulenbelt[9], Tuhina Neogi[10], Maria Popham[11], Carsten O. Schmidt[12], Anushka Soni[13], Ana M. Valdes[11], Najaf Amin[3], Elaine M Dennison[14,15], Niels Eijkelkamp[16], Tamara B. Harris[17], Deborah J. Hart[11], Albert Hofman[3], Frank J.P.M. Huygen[18], Karen A. Jameson[14], Gareth T. Jones[19], Lenore J. Launer[17], Hanneke J.M. Kerkhof[1,2], Marjolein de Kruijf[1,2,18], John McBeth[7], Margreet Kloppenburg[20,21], William E. Ollier[22], Ben Oostra[23], Antony Payton[22], Fernando Rivadeneira[1,2,3], Blair H. Smith[24], Albert V. Smith[5,25], Lisette Stolk[1,2], Alexander Teumer[26], Wendy Thomson[7], André G. Uitterlinden[1,2,3], Ke Wang[10], Sophie H. van Wingerden[3], Nigel K. Arden[14,27], Cyrus Cooper[14,27], David Felson[10], Vilmundur Gudnason[5,25], Gary J Macfarlane[19], Neil Pendleton[8], P. Eline Slagboom[2,9], Tim D. Spector[11], Henry Völzke[12], Annemieke Kavelaars[4&], Cornelia M. van Duijn[3&], Frances M. K. Williams[11&], Joyce B. J. van Meurs[1,2&]

[1] Department of Internal Medicine, Erasmus Medical Center Rotterdam, Rotterdam, The Netherlands
[2] The Netherlands Genomics Initiative-sponsored Netherlands Consortium for Healthy Aging (NGI-NCHA), Leiden/Rotterdam, The Netherlands
[3] Department of Epidemiology, Erasmus Medical Center Rotterdam, Rotterdam, The Netherlands
[4] Laboratory of Neuroimmunology and Developmental Origins of Disease, University Medical Center Utrecht, The Netherlands
[5] Icelandic Heart Association Research Institute, Kopavogur, Iceland
[6] Aberdeen Pain Research Collaboration (Musculoskeletal Research), University of Aberdeen, Aberdeen, UK
[7] Arthritis Research UK Epidemiology Unit, Manchester Academic Health Science Centre, University of Manchester, Manchester, UK
[8] Mental Health and Neurodegeneration Group, School Community Based Medicine, University of Manchester, Manchester, UK
[9] Department of Medical Statistics and Bioinformatics, Section of Molecular Epidemiology, Leiden University Medical Centre, Leiden, The Netherlands
[10] Clinical Epidemiology Unit, Boston University School of Medicine, Boston, Massachusetts, USA
[11] Department of Twin Research and Genetic Epidemiology, King's College London, London, UK
[12] Institute for Community Medicine, University of Greifswald, Greifswald, Germany
[13] NIHR Musculoskeletal Biomedical Research Unit, University of Oxford, Oxford, UK
[14] MRC Lifecourse Epidemiology Unit, University of Southampton, Southampton General Hospital, Southampton, UK
[15] School of Biological Sciences, Victoria University of Wellington, Wellington, New Zealand
[16] Molecular Nociception Group, University College London, London, UK
[17] Intramural Research Program, Laboratory of Epidemiology, Demography, and Biometry, National Institute on Aging, Bethesda, Maryland, USA
[18] Department of Anesthesiology, Erasmus Medical Center Rotterdam, Rotterdam, The Netherlands
[19] Aberdeen Pain Research Collaboration (Epidemiology Group), University of Aberdeen, Aberdeen, UK
[20] Department of Rheumatology, Leiden University Medical Center, Leiden, The Netherlands
[21] Department of Clinical Epidemiology, Leiden University Medical Center, Leiden, The Netherlands
[22] Centre for Integrated Genomic Medical Research, University of Manchester, Manchester, UK
[23] Department of Clinical Genetics, Erasmus Medical Center Rotterdam, Rotterdam, The Netherlands
[24] Medical Research Institute, University of Dundee, Dundee, UK
[25] Department of Medicine, University of Iceland, Reykjavik, Iceland
[26] Institute of Functional Genomics, Ernst Moritz Arndt University Greifswald, University of Greifswald, Greifswald, Germany
[27] NIHR Biomedical Research Unit, Nuffield Department of Orthopaedics, Rheumatology and Musculoskeletal Sciences, University of Oxford, Oxford, UK

** shared first authors*

*[&] shared last authors*

**Corresponding author/address for reprint requests:**

Joyce BJ van Meurs, Genetic Laboratory - Department of Internal Medicine

Room Ee579b, Erasmus MC, PO Box 2040, 3000 CA, Rotterdam, the Netherlands,

Telephone number: +31-10-7038425, e-mail: j.vanmeurs@erasmusmc.nl

**Supplementary Methods:**

**Full description of the Stage 1 GWAS cohorts.**

**The ERF study.** The ERF study (http://www.epib.nl/research/erf/erf_index.html) is a family-based cohort study that is embedded in the Genetic Research in Isolated Populations Program in the southwest of the Netherlands. The aim of this program was to identify genetic risk factors in the development of complex disorders. For the ERF study, 22 families that had at least five children baptized in the community church between 1850 and 1900 were identified with the help of genealogical records. All living descendants of these couples and their spouses were invited to take part in the study. Data collection started in June 2002 and was finished in February 2005. In this study, we focused on 2,347 participants for whom complete phenotypic, genotypic, and genealogical information was available. The medical ethics committee of Erasmus Medical Center Rotterdam approved the study, and informed consent was obtained from all participants. All participants completed a pain homunculus to report the painful sites in the body (pain during at least half of the days during the last six weeks). Individuals were categorised as CWP cases when they report joint pain in the left side of the body, in the right side of the body, above waist, below waist, and in the axial skeleton. Subjects not being a CWP case were categorised as controls, but subjects using pain medication were excluded from the control group.

**The Rotterdam Study.** The Rotterdam Study (www.epib.nl/rotterdamstudy) is a prospective, population based cohort study in the district of Rotterdam, the Netherlands. The initial design of the study is straight-forward: a prospective cohort study among, 7,983 persons living in the well-defined Ommoord district in the city of Rotterdam (78% of 10,215 invitees), called Rotterdam Study I (or RS-I). They were all 55 years of age or over and the oldest participant at the start was 106 years. The study started in the second half of 1989. In 1999, 3,011 participants (out of 4,472 invitees) who had become 55 years of age or moved into the study district since the start of the study were added to the cohort, called Rotterdam Study II (or RS-II). In 2006, a further

extension of the cohort was initiated in which 3,932 subjects were included, aged 45–54 years (out of 6,057 invited), called Rotterdam Study III (RS-III). The participants were all examined in some detail at baseline. They were interviewed at home and then had an extensive set of examinations in a specially built research facility in the centre of their district. These examinations were repeated every 3–4 years in characteristics that could change over time. The participants in the Rotterdam Study are followed for a variety of diseases that are frequent in the elderly. Informed consent was obtained from each participant, and the medical ethics committee of the Erasmus Medical Center Rotterdam approved the study. In Rotterdam, the participants completed the same pain homunculus as the subjects of ERF. Individuals were categorised as CWP cases when they report joint pain in the left side of the body, in the right side of the body, above waist, below waist, and in the axial skeleton. Subjects not being a CWP case were categorised as controls, but subjects using pain medication were excluded from the control group.

**The TwinsUK study.** The TwinsUK cohort (www.twinsuk.ac.uk) is a British adult twin registry shown to be representative of singleton populations and the United Kingdom population. 5687 females aged between the ages of 16-88 completed questionnaires related to chronic widespread pain between 2002 - 2008. These questionnaires asked the participants about any pain in muscles, bones or joints lasting at least one week in the past three months.

## Full description of the Replication Cohorts

**1958BC.** The National Child Development Study, also known as the 1958 British Birth Cohort Study is a large, on-going, prospective cohort study of all children born in England, Scotland, and Wales during one week of March 1958. Detailed methods have been reported previously (Power et al, 2006). Approximately 17,000 participants were recruited at birth and have subsequently been followed up at ages 7, 11, 16, 23, 33, 42 and 45 years. At age 45 years a biomedical survey collected information on health-related factors including the presence of pain.

The sample for the current study was 8,572 individuals who responded to a self-complete pain questionnaire at 45yrs (pain was not joint specific), sent in advance of a nurse interview, and who provided blood samples for genetic analysis.

**The AGES Study.** Age Gene/Environment Susceptibility Reykjavik (AGES-Reykjavik) Study. The Reykjavik Study cohort originally comprised a random sample of 30,795 men and women born in 1907-1935 and living in Reykjavik in 1967. A total of 19,381 people attended, resulting in 71% recruitment rate. The study sample was divided into six groups by birth year and birth date within month. One group was designated for longitudinal follow up and was examined in all stages. One group was designated a control group and was not included in examinations until 1991. Other groups were invited to participate in specific stages of the study. Between 2002 and 2006, the AGES-Reykjavik study re-examined 5764 survivors of the original cohort who had participated before in the Reykjavik Study. Participants came in a fasting state to the clinic and answered questionnaires related to chronic widespread pain. Informed consent was obtained from all participants. Subjects were asked whether they had pain lasting at least one month in the past 12 months. Questions were asked specifically for hand and wrist, hip, knee, shoulder, feet, toes, ankles and back. The AGES Reykjavik Study GWAS was approved by the intra-mural research program of the National Institute on Aging, by the Iceland National Bioethics Committee (VSN: 00-063) and Data Protection Authority.

**The Chingford Study.**

The Chingford Study was established in 1989 when 1003 women, aged 44-67 years, derived from the register of a large general practice in Chingford, North London, were recruited to a prospective population-based longitudinal study of osteoarthritis and osteoporosis. In this study, data on joint and spinal pain, collected as part of the year 4 follow-up visit, was used. Subjects were asked whether they had pain in hand, knee, hip, feet and back during the last year. When they had pain, they were asked for how many days the pain lasted during the last month.

**The DSDBAC Study.** In 1983, 6542 healthy individuals aged between 42 and 92 years old resident in Newcastle and Greater Manchester were recruited into a longitudinal population-based study of cognition in healthy old age. Pain manikin data was collected via postal questionnaire on subjects remaining in the cohort in 2007, and additionally, subjects were asked whether they have had pain for more than 3 months. Pain was not asked joint specific.

**The EPIFUND Study.** EPIFUND is a prospective population-based study of functional disorders. Participants, aged 25-65 years old, were recruited from three primary care registers in the North-west of England. Pain manikin data was collected via a postal questionnaire at baseline and at two follow ups. Additionally to the manikins, subjects were asked whether they have had pain for more than 3 months. Pain was not asked joint specific. DNA was collected using buccal swab sampling from 1189 subjects who participated in all three phases.

**The FOA Study.** The Framingham Osteoarthritis Study is a population-based multigenerational cohort study of over 3500 participants, and is a sub-study of the larger Framingham Heart Study (FHS). In this study, we focused on study participants with information on widespread pain (collected in FOA) and genetic data (collected in FHS). All participants completed a pain homunculus to report the sites in the body having pain, aching, or stiffness on most days. Individuals were categorised as CWP cases when they report joint pain in the left side of the body, in the right side of the body, above waist, below waist, and in the axial skeleton. Subjects not being a CWP case were categorised as controls, but subjects using pain medication were excluded from the control group.

**The GARP Study.** The GARP study from Leiden, the Netherlands, consists of 192 sibling pairs concordant for clinical and radiographically (K/L score) confirmed OA at two or more joint sites among hand, spine (cervical or lumbar), knee or hip[20]. Written informed consent was obtained from each subject as approved by the ethical committees of the Leiden University Medical Center. We recorded pain in the GARP questionnaire by asking the question: Have you

had pain in and around your joints lasting most days of the last month? Patients could choose: hands (left and/ or right), hips (left and/ or right), knees (left and/ or right), back (cervical, thoracic or lumbar region), shoulders (left and/or right) and other sites as specified. When patients indicated that they had pain in the hands, they could specify the locations in the hand in a drawing. When a patient indicated pain in two sections of two contralateral limbs and in the axial skeleton the patient is defined as a case of CWP. For controls, we used 925 randomly chosen Rotterdam Study participants.

**The Hertfordshire Cohort Study (HCS)** is a cohort study of men and women born in Hertfordshire, UK during 1931-39 and still living there in adult life. Approximately 3000 participants were recruited in the late 1990s and have subsequently been followed by clinic visit (East Herts only) and postal clinical outcomes questionnaire (all). The sample for the current study was drawn from individuals who completed a pain questionnaire at using a mannequin to report site of pain, and who had previously provided blood samples for genetic analysis. Individuals were categorised having CWP if they reported having pain for at least three months in a detailed pain questionnaire which corresponded pain in the left and right sides of the body, pain both above and below the waist and back pain (pain was not joint specific). Individuals who did not report such pain but reported use of analgesics were excluded from the analysis. All other individuals were categorized as controls resulting in 90 cases and 2117 controls.

**The SHIP Study.** The SHIP cohort (http://www.medizin.uni-greifswald.de/cm/fv/ship.html) is a prospective, population based cohort study among 4,308 subjects aged ≥20 years from the West Pomerania, Germany. The study was designed to assess prevalence and incidence of risk factors, subclinical disorders and clinical diseases and to investigate associations among them using extensive medical assessments. In this study, we focused on participants for whom complete phenotypic, genotypic, and genealogical information was available. Informed consent was obtained from each participant, and the medical ethics

committee of University of Greifswald approved the study. Subjects were asked to complete questionnaires related to joint pain. These questionnaires asked about pain during the last week, regarding the back, elbow, foot, arms, hands, hip, knee, neck, shoulder, head and facial pain. We decided to exclude head and facial pain, not being joint-related pain. Because no duration was asked for, the pain prevalence in SHIP is one of the highest among the included cohorts.

**Genotyping, Quality Control and Imputation**

The following sample quality control (QC) criteria were applied in the GWAS of RS-I, RS-II, RS-III and ERF: sample call rate >97.5%, gender mismatch with typed X-linked markers, evidence for DNA contamination in the samples using the mean of the autosomal heterozygosity >0.33, exclusion of duplicates or first-degree relatives estimated by pairwise IBD, exclusion of ethnic outliers (>4 SD from population mean using multidimensional scaling (MDS) analysis with 4 principal components (PCs)), and exclusion of samples with missing pain data, age and/or BMI. In the GWAS of TwinsUK, normalised intensity data was pooled, and genotypes were called on the basis of the Illuminus algorithm[1]. No calls were assigned if the most likely call was less than a posterior probability of 0.95. Validation of pooling was done by visual inspection of 100 random, shared SNPs for overt batch effects; none were observed. SNPs that had a low call rate (≤90%), Hardy-Weinberg p-values$<10^{-6}$ and minor allele frequencies < 1% were excluded. Samples with call rates <95% were removed.

Genotype imputation was used to evaluate the association of one and the same SNP across samples typed on different genotyping platforms. Genotypes were imputed for all polymorphic SNPs (minor allele frequency >0.01) using either MACH[2] or IMPUTE[3] software, based upon phased autosomal chromosomes of the HapMap CEU Phase II panel (release 22, build 36), orientated on the positive strand. Imputation QC metrics from MACH and IMPUTE were used for filtering out SNPs with low-quality data.

**Stage 1 GWAS Meta-Analysis**

The estimated inflation factors were 1.176, 1.014, 1.008, 1.006, and 0.989 for ERF, RS-I, RS-II, RS-III, and TwinsUK respectively. SNPs with a minor allele frequency <0.05, a MACH $r^2$-hat <0.30, or a SNPTEST proper_info <0.40 were excluded from the meta-analysis. We obtained the combined results of the 2,224,068 autosomal SNPs, pooling the effect sizes by means of a fixed effects inverse variance meta-analysis as implemented in METAL. Estimated heterogeneity variance and forest plots were generated using the Comprehensive Meta-Analysis[4] software. Regional association plots of the meta-analysis results were obtained with LocusZoom[5].

**Sequenom iPLEX and Taqman Allelic Discrimination genotyping**

Genotypes for CHINGFORD, EPIFUND, and HCS were generated using Sequenom iPLEX genotyping and Taqman Allelic Discrimination genotyping. Genomic DNA was extracted from samples of peripheral venous blood according to standard procedures. 1-2 ng genomic DNA was dispensed into 384-wells plates using a Caliper Sciclone ALH3000 pipetting robot (Caliper LS, Mountain View, CA, USA).

For Sequenom iPLEX genotyping, multiplex PCR assays were designed using Assay Designer on the website (https://mysequenom.com/tools/genotyping/default.aspx). For this, sequences containing the SNP site and at least 100 bp of flanking sequence on either side of the SNP were used. Briefly, 2 ng genomic DNA was amplified in a 5 ul reaction containing 1 × Taq PCR buffer (Sequenom), 2 mM $MgCl_2$, 500 uM each dNTP, 100 nM each PCR primer, 0.5 U Taq (Sequenom). The reaction was incubated at 94°C for 4 minutes followed by 45 cycles of 94°C for 20 seconds, 56°C for 30 seconds, 72°C for 1 minute, followed by 3 minutes at 72°C. Excess dNTPs were then removed from the reaction by incubation with 0.3 U shrimp alkaline phosphatase (Sequenom) at 37°C for 40 minutes followed by 5 minutes at 85°C to deactivate the enzyme. Single primer extension over the SNP was carried out in a final concentration of between 0.731

uM and 1.462 uM for each extension primer (depending on the mass of the probe), iPLEX termination mix (Sequenom), 10x iPLEX Buffer Plus and iPLEX enzyme (Sequenom) and cycled using the following program; 94°C for 30 seconds followed by 94°C for 5 seconds, 5 cycles of 52°C for 5 seconds, and 80°C for 5 seconds, the last three steps were repeated 40 times, then 72°C for 3 minutes. The reaction was then desalted by addition of 6 mg clear resin (Sequenom) followed by mixing (15 minutes) and centrifugation (5 min, 3,000rpm) to settle the contents of the tube. The extension product was then spotted onto a 384 well spectroCHIP using the SEQUENOM MassARRAY Nanodispenser RS1000 before analysis on the MassARRAY Compact System (Sequenom). Data collection was performed using SpectroACQUIRE 3.3.1.13 and clustering was called using TYPER Analyzer 4.0.3.18 (Sequenom). Additionally to ensure data quality genotypes for each subject were also checked manually.

For Taqman Allelic Discrimination genotyping (Applied Biosystems Inc., Foster City, CA, USA), all SNP assays were available at www.appliedbiosystems.com as pre-designed assays. The PCR reaction mixture included 1-2 ng of genomic DNA in a 2 µl volume and the following reagents: FAM and VIC probes (200 nM), primers (0.9 uM), 2x Taqman PCR master mix (Applied Biosystems Inc., Foster City, CA, USA). Reagents were dispensed in a 384-well plate using the Deerac Equator NS808 (Deerac Fluidics, Dublin, Ireland). PCR cycling reaction were performed in 384 wells PCR plates in an ABI 9700 PCR system (Applied Biosystems Inc., Foster City, CA, USA) and consisted of initial denaturation for 15 minutes at 95° C, and 40 cycles with denaturation of 15 seconds at 95° C and annealing and extension for 60 seconds at 60° C. Results were analysed by the ABI Taqman 7900HT using the sequence detection system 2.22 software (Applied Biosystems Inc., Foster City, CA, USA).

**RNA isolation and real-time PCR for mRNA quantitation**

Total RNA was isolated with the Trizol (Invitrogen, Paisley, UK) method and 1 µg of total RNA was used to synthesize cDNA with SuperScript Reverse Transcriptase (Invitrogen;

carrageenan experiment) or iScript$^{TM}$ Select cDNA Synthesis Kit (Invitrogen; CFA experiment) using random hexamers.

Using quantitative PCR, mRNA levels of Cct5 and Fam173b were measured in the spinal cord and the DRG. The real-time PCR reaction with SYBR green Master mix (Bio-Rad, Alphen aan den Rijn, the Netherlands) was performed on the iQ5 Real-Time PCR Detection System (BioRad; carrageenan experiment) or Mastercycler ep realplex (Eppendorf; CFA experiment). For both experiments, the gene expression levels were normalized for Gapdh and β-actin expression levels (housekeeping genes). We used the following primers:

- Cct5 forward: GTCTCATGGGGCTTGAGG, reverse: GTCCGCATTGTGTTTGCTAC.
- Fam173b forward: TGGTGTGCCCCAGATGAT, reverse: TGCCCTCTCCAGTGGTGT.
- Gapdh forward: TGAAGCAGGCATCTGAGGG, reverse: CGAAGGTGGAAGAGTGGGAG.
- β-actin forward: AGAGGGAAATCGTGCGTGAC, reverse: CAATAGTGATGACCTGGCCGT.

We designed the primers to be intron-spanning thereby targeting the first two exons of Cct5 and the last two exons of Fam173b. The thermocycling profile of amplification was 10 min at 95°C, 40 cycles of 15s at 95°C and 1 min at 60°C, 1 min at 95°C, and 2 min at 65°C, followed by a final meltcurve analysis.

**Supplementary Figures and Tables:**

**Figure S1A.** Heat withdrawal latency time measurements at day 0 and day 6 after intraplantar carrageenan (n=4) or saline (n=4) injection. The latency time was measured using the Hargreaves Test. Data are expressed as means ± SEM. *** = $p<0.001$.

**Figure S1B.** Heat withdrawal latency time measurements at day 0, day 1, and day 3 after intraplantar Complete Freund's Adjuvant (CFA) (n=4) or saline (n=4) injection. The latency time was measured using the Hargreaves Test. Data are expressed as means ± SEM. *** = $p<0.001$.

**Table S1.** Pain assessment methods according to the different studies.

| Study | Method | Areas of the body checked | | | | | | | | | Duration of pain* |
|---|---|---|---|---|---|---|---|---|---|---|---|
| | | *Back* | *Elbow* | *Foot* | *Hand* | *Hip* | *Knee* | *Neck* | *Shoulder* | *Others* | |
| ***Stage 1*** | | | | | | | | | | | |
| ERF study | Homunculus (drawing circles) | yes | yes | yes | yes | yes | yes | yes | yes | no | > half of the days during last 6 weeks |
| RS-I | Homunculus (drawing circles) | yes | yes | yes | yes | yes | yes | yes | yes | no | > half of the days during last 6 weeks |
| RS-II | Homunculus (drawing circles) | yes | yes | yes | yes | yes | yes | yes | yes | no | > half of the days during last 6 weeks |
| RS-III | Homunculus (drawing circles) | yes | yes | yes | yes | yes | yes | yes | yes | no | > half of the days during last 6 weeks |
| TWINSUK | Questionnaire | yes ± | no ± | yes ± | yes ± | no ± | no ± | yes ± | yes ± | arm, leg, chest± | > 1 week during the last 3 months |
| ***Stage 2a*** | | | | | | | | | | | |
| 1958BC | Homunculus (shading areas) | yes ± | yes ± | yes ± | yes ± | yes ± | yes ± | yes ± | yes ± | yes ± | >=3 months |
| AGES | Questionnaire | yes | no | yes, (not s.s.)° | yes,(not s.s.)° | yes | yes | no | yes | no | > 1 month during last year |
| DSDBAC | Homunculus (shading areas) | yes ± | yes ± | yes ± | yes ± | yes ± | yes ± | yes ± | yes ± | yes ± | >=3 months |
| FOA | Homunculus (shading areas) | yes | yes | yes (incl. indiv. joints)# | yes (incl. indiv. joints)# | yes | yes | yes | yes | ankle, wrist | "pain on most days" |
| GARP | Questionnaire | yes | yes | no | yes | yes | yes | yes | no | spine | not asked |
| SHIP | Questionnaire | yes ± | yes ± | yes ± | yes ± | yes ± | yes ± | yes ± | yes ± | head, face, arm, abdomen, pelvic ± | not asked |
| ***Stage 2b*** | | | | | | | | | | | |
| CHINGFORD | Joint Symptom Questionnaire | yes | no | no | yes | yes | yes | yes | no | no | not asked |
| EPIFUND | Homunculus (shading areas) | yes ± | yes ± | yes ± | yes ± | yes ± | yes ± | yes ± | yes ± | yes ± | >=3 months |
| HCS | Homunculus (shading areas) | yes ± | yes ± | yes ± | yes ± | no ± | yes ± | yes ± | no ± | upper arm, lower arm, upper leg, lower leg, sternum, chest, abdomen, buttock ± | "pain lasting most days of the month over the last year" – extra question: pain lasted > 3 months? |

* If available, the duration of pain criteria was used; # Incl. indiv. joints: the individual joints were scored; ° Not s.s.: information about this joint was not side specific. ± Not joint specific.

ERF study = Erasmus Rucphen Family study; RS = Rotterdam Study; TwinsUK = The UK Adult Twin Registry; 1958BC = 1958 Birth Cohort; AGES = Age, Gene/Environment Susceptibility study Reykjavik; CHINGFORD = Chingford 1000 Women Study; DSDBAC = Dyne Steel DNA Bank for Ageing and Cognition; EPIFUND = EPIdemiological study of FUNctional Disorders study; FOA = Framingham Osteoarthritis Study; GARP = Genetics OsteoArthritis and Progression study Leiden; HCS = Hertfordshire Cohort Study. SHIP = Study of Health In Pomerania.

**Table S2.** Study design, number of cases and controls, and sample quality control for the Stage 1 GWAS cohorts.

| **Study** | | **Study design** | **Total sample size (N)** | **Sample QC** | | **Number of samples in the analyses** | **References** |
|---|---|---|---|---|---|---|---|
| **Short name** | **Full name** | | | **Call rate** | **Other exclusions** | | |
| ERF study | Erasmus Rucphen Family study | Family Based Cohort | 2300 | > 95% | 1) Excess heterozygosity based on FDR<br>2) Ethnic outliers<br>3) Gender mismatch<br>4) Missing phenotype | 149 cases,<br>665 controls | [6] |
| RS-I | Rotterdam Study I | Population Based Cohort | 7983 | ≥ 97.5% | 1) Gender mismatch with typed Xlinked markers;<br>2) Excess autosomal heterozygosity > 0.336~FDR>0.1%;<br>3) Duplicates and/or 1st or 2nd degree relatives using IBS probabilities > 97% from PLINK;<br>4) Ethnic outliers using IBS distances > 3SD from PLINK;<br>5) Missing pain, age, and BMI information. | 563 cases,<br>1892 controls | [7] |
| RS-II | Rotterdam Study II | Population Based Cohort | 3011 | ≥ 97.5% | 1) Gender mismatch with typed X-linked markers;<br>2) Excess autosomal heterozygosity (F<-0.055);<br>3) Duplicates and/or 1st degree relatives using IBD piHAT >40% from PLINK;<br>4) Ethnic outliers using IBS distances > 4SD mean HapMap CEU cluster from PLINK;<br>5) Missing pain, age, and BMI information. | 110 cases,<br>668 controls | [7] |
| RS-III | Rotterdam Study III | Population Based Cohort | 3932 | ≥ 97.5% | 1) Gender mismatch with typed Xlinked markers;<br>2) Excess autosomal heterozygosity (F<-0.055);<br>3) Duplicates and/or 1st degree relatives using IBD piHAT >40% from PLINK;<br>4) Ethnic outliers IBS distances > 4SD mean HapMap CEU cluster from PLINK;<br>5) Missing pain, age, and BMI information. | 85 cases,<br>868 controls | [7] |
| TwinsUK | UK Adult Twin Registry | Twins Based Cohort | 5687 | ≥ 95% | 1) Heterozygosity <33% or >67%;<br>2) Ethnic outliers;<br>3) Related individuals and duplicates;<br>4) Missing pain, age, and BMI information. | 401 cases,<br>1698 controls | [8, 9] |

ERF study = Erasmus Rucphen Family study; RS = Rotterdam Study; TwinsUK = The UK Adult Twin Registry.

**Table S3.** Information on genotyping methods, quality control of SNPs, and imputation for the Stage 1 GWAS cohorts.

| **Cohort** | **Genotyping** | | | | | | **Imputation** | | |
|---|---|---|---|---|---|---|---|---|---|
| | **Platform** | **Genotype Calling Algorithm** | **Inclusion Criteria** | | | **SNPs that met QC criteria** | **Imputation Software** | **Inclusion Criteria** | |
| | | | **MAF** | **Callrate** | **p-value for HWE** | | | **MAF** | **Imputation Quality Score** |
| ERF study | Illumina 318K, 370K, Affymetrix 250K | BRLMM, BeadStudio | >0.5% | >95% | $>10^{-06}$ | NA | MACH | >0% | $r^2$-hat ≥ 0.30 |
| RS-I | Illumina / HumanHap 550K V.3, Illumina / HumanHap 550K V.3 DUO; | BeadStudio Genecall | ≥1% | ≥97.5% | $>10^{-06}$ | 512,349 | MACH | ≥0% | (O/E)σ2 ratio≥0.1 $r^2$-hat ≥ 0.30 |
| RS-II | Illumina / HumanHap 550 V.3 DUO; Illumina / HumanHap 610 QUAD | Genomestudio Genecall | ≥1% | ≥97.5% | $>10^{-06}$ | 466,389 | MACH | ≥1% | (O/E)σ2 ratio≥0.1 $r^2$-hat ≥ 0.30 |
| RS-III | Illumina / HumanHap 610 QU | Genomestudio Genecall | ≥1% | ≥97.5% | $>10^{-06}$ | 514,073 | MACH | ≥1% | (O/E)σ2 ratio≥0.1 $r^2$-hat ≥ 0.30 |
| TwinsUK | Illumina / HumanHap 300 & 550 | Illuminus | ≥1% | ≥95.0% | $>10^{-06}$ | 295,702 | IMPUTE | >0% | proper-info ≥ 0.40 |

ERF study = Erasmus Rucphen Family study; RS = Rotterdam Study; TwinsUK = The UK Adult Twin Registry.

**Table S4.** Study design, number of cases and controls, and sample quality control for the Stage 2 cohorts.

| Study | | Study design | Total sample size (N) | Sample QC | | Number of samples in the analyses | References |
|---|---|---|---|---|---|---|---|
| Short name | Full name | | | Call Rate | Other exclusions | | |
| 1958BC | National Child Development (1958 Birth Cohort) Study | Prospective Birth Cohort | 4958 | ≥97% | 1) Autosomal heterozygosity<br>2) Non-Caucasian<br>3) Average difference in probe intensities across SNPs<br>4) Individuals with >5% IBD<br>5) Gender mismatch<br>6) >10% discordance upon repeated genotyping<br>7) Missing phenotype | 315 cases, 2206 controls | [10, 11] |
| AGES | Age, Gene Environment Susceptibility Reykjavik Study | Population Based Cohort | 3219 | ≥97% | 1) Sample failure<br>2) Genotype mismatch with reference panel<br>3) Sex mismatch | 173 cases, 1204 controls | [12] |
| DSDBAC | Dyne Steel DNA Bank for Ageing and Cognition | Population Based Cohort | 6542 | >95% | 1) Gender mismatch<br>2) IBD sharing >0.25<br>3) Non-Caucasians by multi-dimensional scaling | 81 cases, 219 controls | [13] |
| FOA | The Framingham Osteoarthritis Study | Population Based Cohort | 4792 | ≥97% | 1) Subject heterozygosity > ± 5 SDs from the the mean<br>2) Missing pain, age, and BMI information | 384 cases, 814 controls | [14] |
| GARP | Genetics osteoARthritis and Progression Study | Case Based Cohort | 384 | >99% | NA | 67 cases, 925 RS controls | [15] |
| SHIP | Study of Health In Pomerania | Population Based Cohort | 4081 | >92% | 1) Duplicate samples (by IBS)<br>2) Reported/genotyped gender mismatches | 183 cases, 589 controls | [16, 17] |
| CHINGFORD | Chingford Study | Population Based Cohort | 831 | NA | 1) Missing phenotype information. | 48 cases, 337 controls | [18, 19] |
| EPIFUND | EPIdemiological study of FUNctional Disorders | Population Based Cohort | 6290 | >95% | None | 139 cases, 503 controls | [20] |
| HCS | Hertfordshire Cohort Study | Population Based Cohort | 1073 | >95% | Missing phenotype information. | 90 cases, 2117 controls | [21] |

1958BC = 1958 Birth Cohort; AGES = Age, Gene/Environment Susceptibility study Reykjavik; CHINGFORD = Chingford 1000 Women Study; DSDBAC = Dyne Steel DNA Bank for Ageing and Cognition; EPIFUND = EPIdemiological study of FUNctional Disorders study; FOA = Framingham Osteoarthritis Study; GARP = Genetics OsteoArthritis and Progression study Leiden; HCS = Hertfordshire Cohort Study. SHIP = Study of Health In Pomerania.

**Table S5.** Information on genotyping methods, quality control of SNPs, and imputation for the Stage 2 cohorts.

| Cohort | Genotyping | | | | | | Imputation | | |
|---|---|---|---|---|---|---|---|---|---|
| | Platform | Genotype Calling Algorithm | Inclusion Criteria | | | SNPs that met our replication QC criteria | Imputation Software | Inclusion Criteria | |
| | | | MAF | Callrate | p-value for HWE | | | MAF | Imputation Quality Score |
| 1958BC | Affymetrix v6.0 (WTCCC2), Illumina 1.2M chip (WTCCC2), and Illumina 550k (T1DGC) | Chiamo software (adapted for Affymetrix 6.0 SNP data) and Illuminus | >5% | ≥ 98% | >0.05 | 2 SNPs + 7 proxy SNPs | NA | NA | NA |
| AGES | Illumina 370 CNV BeadChip | BeadStudio | ≥ 1% | ≥97% | $>10^{-6}$ | 10 | MACH | >0% | r2-hat ≥ 0.30 |
| DSDBAC | Illumina610-Quadv1 chip | Genomestudio | ≥1% | ≥98% | $>10^{-3}$ | 10 | MACH | >0% | $r^2$-hat ≥ 0.30 |
| FOA | Affymetrix 500K and 50K supplemental array | BRLMM | ≥1% | ≥97% | $\geq 10^{-6}$ | 10 | MACH | ≥1% | r2-hat ≥ 0.3 |
| GARP | Illumina Human660W | Genome studio | >5% | >98% | >0.001 | 10 | IMPUTE | >5% | proper-info ≥ 0.85 |
| SHIP | Affymetrix SNP Array 6.0 | Birdseed2 | ≥ 0% | ≥ 0% | ≤ 1 | 10 | IMPUTE v0.5.0 | ≥ 0% | proper_info ≤ 1 |
| CHINGFORD | Sequenom iPLEX and Taqman Allelic Discrimination | Taqman / Sequenom | NA | ≥95% | >0.05 | 10 | NA | NA | NA |
| EPIFUND | Sequenom iPLEX | Sequenom | NA | ≥95% | >0.05 | 1 SNP + 1 proxy SNP | NA | NA | NA |
| HCS | Taqman Allelic Discrimination | Taqman | NA | NA | NA | 5 | NA | NA | NA |

1958BC = 1958 Birth Cohort; AGES = Age, Gene/Environment Susceptibility study Reykjavik; CHINGFORD = Chingford 1000 Women Study; DSDBAC = Dyne Steel DNA Bank for Ageing and Cognition; EPIFUND = EPIdemiological study of FUNctional Disorders study; FOA = Framingham Osteoarthritis Study; GARP = Genetics OsteoArthritis and Progression study Leiden; HCS = Hertfordshire Cohort Study. SHIP = Study of Health In Pomerania.

**Table S6.** Genotyped SNPs for replication**.**

| **Study** | rs13361160 | rs12132674 | rs7680363 | rs7835968 | rs2249104 | rs17796312 | rs4837492 | rs11606304 | rs524513 | rs8065610 |
|---|---|---|---|---|---|---|---|---|---|---|
| 1958BC | P: rs1508850 | P: rs2843016 | P: rs12511202 | P: rs7830100 | P: rs3858511 | P: rs12609590 | G | NG | P: rs3895875 | G |
| AGES | I | I | I | I | I | I | I | I | I | G |
| CHINGFORD | G | G | G | G | G | G | G | G | G | G |
| DSDBAC | I | I | I | I | I | I | G | I | I | G |
| EPIFUND | G | P: rs2843016 | NG | NG | NG | NG | NG | NG | NG | NG |
| FOA | I | I | I | I | I | I | I | I | I | I |
| GARP | I | I | I | I | I | I | G | I | I | G |
| HCS | G | G | G | NG | NG | NG | NG | G | NG | G |
| SHIP | I | I | G | I | I | I | G | I | I | G |

G = Genotyped, I = Imputed, NG = Not Genotyped, P = Proxy Genotyped. The rs-number called behind P gives the used proxy.

1958BC = 1958 Birth Cohort; AGES = Age, Gene/Environment Susceptibility study Reykjavik; CHINGFORD = Chingford 1000 Women Study; DSDBAC = Dyne Steel DNA Bank for Ageing and Cognition; EPIFUND = EPIdemiological study of FUNctional Disorders study; FOA = Framingham Osteoarthritis Study; GARP = Genetics OsteoArthritis and Progression study Leiden; HCS = Hertfordshire Cohort Study. SHIP = Study of Health In Pomerania.

**Table S7.** Mean age and BMI for all participating studies

| Cohort | Mean and Standard Deviation of the covariates age and BMI | | | | | |
|---|---|---|---|---|---|---|
| | Age (y) | | | BMI (kg/m²) | | |
| | Cases | Controls | P-value Cases vs. Controls | Cases | Controls | P-value Cases vs. Controls |
| ERF study | 51.4 (+/- 11.2) | 45.3 (+/- 14.3) | 1.10E-06 | 27.1 (+/- 5.1) | 26.1 (+/- 4.6) | 1.30E-02 |
| RS-I | 68.9 (+/- 8.6) | 69.5 (+/- 9.0) | 1.48E-01 | 27.4 (+/- 4.3) | 26.6 (+/- 4.1) | 1.00E-06 |
| RS-II | 67.2 (+/- 7.6) | 68.0 (+/- 7.5) | 2.66E-01 | 29.0 (+/- 5.0) | 27.7 (+/- 4.3) | 3.88E-03 |
| RS-III | 57.8 (+/- 7.8) | 56.2 (+/- 5.8) | 1.85E-02 | 28.6 (+/- 5.2) | 27.4 (+/- 5.0) | 4.23E-02 |
| TwinsUK | 55.4 (+/- 11.6) | 51.1 (+/- 13.9) | 6.50E-10 | 26.6 (+/- 5.4) | 24.7 (+/- 4.3) | 2.70E-11 |
| | | | | | | |
| 1958BC | NA | NA | NA | 27.7 (+/- 6.1) | 26.7 (+/- 5.3) | 4.00E-3 |
| AGES | 75.7 (+/- 5.2) | 76.6 (+/- 5.6) | 6.36E-1 | 28.6 (+/- 4.8) | 27.2 (+/- 4.8) | 1.44E-5 |
| DSDBAC | 79.7 (+/-4.7) | 80.3 (+/-5.7) | 4.09E-1 | NA | NA | NA |
| FOA | 61.0 (+/- 11.3) | 58.5 (+/- 13.6) | 2.00E-3 | 28.6 (+/- 5.8) | 25.6 (+/- 4.8) | 2.00E-16 |
| GARP | 58.5 (+/-6.9) | 57.7 (+/- 1.4) | 7.00E-3 | 28.1 (+/- 6.1) | 26.3 (+/- 5.9) | 1.40E-2 |
| SHIP | 60.7 (+/- 12.9) | 56.7 (+/- 13.4) | 1.30E-3 | 29.3 (+/-5.41) | 27.6 (+/- 5.136) | 5.00E-5 |
| CHINGFORD | 57.2 (+/-5.9) | 56.5 (+/-8.7) | 5.89E-1 | 27.1 (+/-4.6) | 26.3 (+/-4.5) | 3.29E-1 |
| EPIFUND | 50.9 (+/-8.8) | 48.5 (+/-10.4) | 1.37E-2 | NA | NA | NA |
| HCS | 66.8 (+/- 2.7) | 66.4 (+/-2.8) | 4.88E-1 | 29.7 (+/- 6.1) | 26.8 (+/- 4.5) | 1.69E-5 |

ERF study = Erasmus Rucphen Family study; RS = Rotterdam Study; TwinsUK = The UK Adult Twin Registry; 1958BC = 1958 Birth Cohort; AGES = Age, Gene/Environment Susceptibility study Reykjavik; CHINGFORD = Chingford 1000 Women Study; DSDBAC = Dyne Steel DNA Bank for Ageing and Cognition; EPIFUND = EPIdemiological study of FUNctional Disorders study; FOA = Framingham Osteoarthritis Study; GARP = Genetics OsteoArthritis and Progression study Leiden; HCS = Hertfordshire Cohort Study. SHIP = Study of Health In Pomerania.

**Table S8.** Information on statistical analysis for the Stage 1 GWAS cohorts.

| Cohort | Association Analyses | | |
|---|---|---|---|
| | **SNPs in meta-analysis** | **Analysis Software** | **References Analysis Software** |
| ERF study | 2,463,846 | ProbABEL | [22] |
| RS-I | 2,542,336 | GRIMP (MACH2DAT) | [2, 23] |
| RS-II | 2,536,671 | GRIMP (MACH2DAT) | [2, 23] |
| RS-III | 2,533,563 | GRIMP (MACH2DAT) | [2, 23] |
| TwinsUK | 2,460,943 | PLINK | [24] |

ERF study = Erasmus Rucphen Family study; RS = Rotterdam Study; TwinsUK = The UK Adult Twin Registry.

**Table S9:** 29 proxy SNPs which are in LD with rs13361160 ($r^2$>0.1).

| SNP | Proxy | Distance | $r^2$ | D' | Gene Variant | GeneName | Possible eQTL-effect? |
|---|---|---|---|---|---|---|---|
| rs13361160 | rs1045392 | 56655 | 0.216 | 0.892 | 3'UTR | FAM173B | |
| rs13361160 | rs1045369 | 57016 | 0.216 | 0.892 | 3'UTR | FAM173B | |
| rs13361160 | rs17294394 | 58695 | 0.105 | 0.329 | Intronic | FAM173B | |
| rs13361160 | rs4557374 | 59018 | 0.144 | 1.000 | Intronic | FAM173B | |
| rs13361160 | rs7716217 | 59785 | 0.216 | 0.892 | Intronic | FAM173B | |
| rs13361160 | rs7716565 | 60006 | 0.135 | 1.000 | Intronic | FAM173B | |
| rs13361160 | rs7716851 | 60151 | 0.144 | 1.000 | Intronic | FAM173B | |
| rs13361160 | rs7736719 | 60169 | 0.144 | 1.000 | Intronic | FAM173B | |
| rs13361160 | rs6887347 | 61868 | 0.215 | 1.000 | Intronic | FAM173B | |
| rs13361160 | rs16884328 | 62099 | 0.109 | 0.534 | Intronic | FAM173B | |
| rs13361160 | rs6887590 | 62124 | 0.144 | 1.000 | Intronic | FAM173B | |
| rs13361160 | rs6888157 | 62153 | 0.144 | 1.000 | Intronic | FAM173B | |
| rs13361160 | rs6880482 | 64235 | 0.144 | 1.000 | Intronic | FAM173B | |
| rs13361160 | rs2292264 | 65667 | 0.186 | 1.000 | Intronic | FAM173B | |
| rs13361160 | rs7710415 | 65930 | 0.135 | 1.000 | Intronic | FAM173B | |
| rs13361160 | rs16884348 | 66129 | 0.154 | 1.000 | Intronic | FAM173B | yes: $r^2$ with eQTL-SNP rs2445871 = 0.872 |
| rs13361160 | rs12653481 | 67213 | 0.186 | 1.000 | Intronic | FAM173B | |
| rs13361160 | rs2438652 | 69438 | 0.165 | 1.000 | Non-synonymous-coding | FAM173B | yes: $r^2$ with eQTL-SNP rs2445871 = 0.818 |
| rs13361160 | rs2445871 | 70170 | 0.135 | 1.000 | Intronic | FAM173B | yes: rs2445871 is an eQTL SNP |
| rs13361160 | rs2607326 | 70968 | 0.134 | 0.851 | Intronic | FAM173B | |
| rs13361160 | rs2607328 | 71623 | 0.125 | 1.000 | Intronic | FAM173B | |
| rs13361160 | rs2607298 | 82296 | 0.104 | 0.639 | Intronic | CCT5 | yes: $r^2$ with eQTL-SNP rs2244964 = 0.953 |
| rs13361160 | rs2445867 | 83341 | 0.104 | 0.639 | Intronic | CCT5 | yes: $r^2$ with eQTL-SNP rs2244964 = 0.953 |
| rs13361160 | rs1042392 | 86338 | 0.159 | 0.854 | Synonymous coding | CCT5 | yes: $r^2$ with eQTL-SNP rs2244964 = 0.904 |
| rs13361160 | rs2028274 | 86787 | 0.104 | 0.639 | Intronic | CCT5 | yes: $r^2$ with eQTL-SNP rs2244964 = 0.953 |
| rs13361160 | rs2028272 | 86899 | 0.111 | 0.646 | Intronic | CCT5 | yes: $r^2$ with eQTL-SNP rs2244964 = 0.909 |
| rs13361160 | rs7710938 | 87070 | 0.123 | 0.732 | Intronic | CCT5 | yes: $r^2$ with eQTL-SNP rs2244964 = 0.951 |
| rs13361160 | rs7729006 | 87146 | 0.123 | 0.732 | Intronic | CCT5 | yes: $r^2$ with eQTL-SNP rs2244964 = 0.951 |
| rs13361160 | rs2438653 | 89550 | 0.104 | 0.639 | Intronic | CCT5 | yes: $r^2$ with eQTL-SNP rs2244964 = 0.953 |

**Table S10.** Association results of 92 candidate SNPs in the stage 1 GWAS meta-analysis sorted by gene names and P-values. The strongest associated SNPs (p<0.01) were shaded (P<0.01).

| MarkerName | Gene | Allele1 | Allele2 | Freq A1 | OR | 95%CI | P-value |
|---|---|---|---|---|---|---|---|
| **rs2020917** | **COMT** | **t** | **c** | **0.266** | **0.856** | **(0.772-0.948)** | **2.97E-03** |
| **rs5993883** | **COMT** | **t** | **g** | **0.496** | **1.142** | **(1.046-1.247)** | **3.05E-03** |
| rs5746846 | COMT | c | g | 0.501 | 1.098 | (1.005-1.200) | 3.85E-02 |
| rs1544325 | COMT | a | g | 0.457 | 1.087 | (0.995-1.187) | 6.38E-02 |
| rs2239393 | COMT | a | g | 0.603 | 1.081 | (0.986-1.184) | 9.51E-02 |
| rs4818 | COMT | c | g | 0.604 | 1.081 | (0.986-1.184) | 9.66E-02 |
| rs4646312 | COMT | t | c | 0.604 | 1.078 | (0.984-1.181) | 1.07E-01 |
| rs165728 | COMT | t | c | 0.935 | 0.812 | (0.615-1.071) | 1.41E-01 |
| *rs4680* | *COMT* | *a* | *g* | *0.532* | *1.057* | *(0.963-1.162)* | *2.44E-01* |
| rs4633 | COMT | t | c | 0.536 | 1.048 | (0.957-1.147) | 3.09E-01 |
| rs174699 | COMT | t | c | 0.929 | 0.860 | (0.632-1.172) | 3.41E-01 |
| rs2097903 | COMT | a | t | 0.500 | 0.961 | (0.875-1.056) | 4.08E-01 |
| rs165774 | COMT | a | g | 0.313 | 1.034 | (0.933-1.146) | 5.21E-01 |
| rs5993882 | COMT | t | g | 0.768 | 1.027 | (0.924-1.143) | 6.20E-01 |
| rs174674 | COMT | a | g | 0.274 | 1.020 | (0.917-1.134) | 7.19E-01 |
| | | | | | | | |
| **rs10483639** | **GCH1** | **c** | **g** | **0.203** | **0.846** | **(0.755-0.948)** | **3.84E-03** |
| **rs752688** | **GCH1** | **t** | **c** | **0.202** | **0.846** | **(0.755-0.948)** | **3.94E-03** |
| **rs4411417** | **GCH1** | **t** | **c** | **0.798** | **1.181** | **(1.055-1.323)** | **4.00E-03** |
| rs8007267 | GCH1 | t | c | 0.178 | 0.890 | (0.790-1.001) | 5.23E-02 |
| rs3783641 | GCH1 | a | t | 0.192 | 0.902 | (0.803-1.013) | 8.15E-02 |
| | | | | | | | |
| **rs599548** | **OPRM1** | **a** | **g** | **0.142** | **1.189** | **(1.050-1.346)** | **6.41E-03** |
| rs558025 | OPRM1 | a | g | 0.739 | 1.085 | (0.979-1.203) | 1.19E-01 |
| rs1799971 | OPRM1 | a | g | 0.873 | 0.907 | (0.796-1.033) | 1.42E-01 |
| rs563649 | OPRM1 | t | c | 0.091 | 1.088 | (0.934-1.268) | 2.78E-01 |
| rs2075572 | OPRM1 | c | g | 0.551 | 1.051 | (0.957-1.154) | 3.00E-01 |
| | | | | | | | |
| rs10485171 | CNR1 | a | g | 0.530 | 1.104 | (1.009-1.207) | 3.09E-02 |
| rs1078602 | CNR1 | a | g | 0.485 | 0.941 | (0.861-1.030) | 1.88E-01 |
| rs6454674 | CNR1 | t | g | 0.705 | 0.957 | (0.869-1.055) | 3.81E-01 |
| | | | | | | | |
| rs2400707 | ADRB2 | a | g | 0.444 | 0.909 | (0.831-0.994) | 3.68E-02 |
| rs1042714 | ADRB2 | c | g | 0.548 | 1.117 | (1.000-1.246) | 4.90E-02 |
| rs12654778 | ADRB2 | a | g | 0.367 | 1.072 | (0.978-1.175) | 1.39E-01 |
| rs1042713 | ADRB2 | a | g | 0.363 | 1.070 | (0.976-1.173) | 1.48E-01 |
| rs17778257 | ADRB2 | a | t | 0.630 | 0.941 | (0.859-1.032) | 1.96E-01 |
| rs1042718 | ADRB2 | a | c | 0.162 | 1.027 | (0.912-1.156) | 6.62E-01 |

| MarkerName (II) | Gene | Allele1 | Allele2 | Freq A1 | OR | 95%CI | P-value |
|---|---|---|---|---|---|---|---|
| rs8192619 | TAAR1 | a | g | 0.056 | 0.732 | (0.517-1.035) | 7.77E-02 |
| | | | | | | | |
| rs12584920 | HTR2A | t | g | 0.187 | 0.903 | (0.804-1.015) | 8.75E-02 |
| rs4941573 | HTR2A | a | g | 0.578 | 0.975 | (0.891-1.066) | 5.72E-01 |
| rs6313 | HTR2A | a | g | 0.419 | 1.019 | (0.932-1.114) | 6.77E-01 |
| rs17289394 | HTR2A | a | g | 0.400 | 0.992 | (0.906-1.085) | 8.55E-01 |
| | | | | | | | |
| rs10502058 | GRIA4 | t | c | 0.884 | 0.888 | (0.774-1.019) | 9.18E-02 |
| rs2510177 | GRIA4 | a | g | 0.090 | 1.140 | (0.976-1.331) | 9.72E-02 |
| rs10895837 | GRIA4 | t | c | 0.116 | 1.122 | (0.977-1.287) | 1.03E-01 |
| rs642544 | GRIA4 | t | g | 0.583 | 0.960 | (0.878-1.051) | 3.78E-01 |
| rs17104711 | GRIA4 | a | g | 0.084 | 1.039 | (0.887-1.217) | 6.38E-01 |
| | | | | | | | |
| rs8065080 | TRPV1 | t | c | 0.618 | 0.929 | (0.848-1.017) | 1.09E-01 |
| | | | | | | | |
| rs1143623 | IL-1B | c | g | 0.721 | 0.923 | (0.836-1.019) | 1.12E-01 |
| rs16944 | IL-1B | a | g | 0.344 | 1.076 | (0.980-1.182) | 1.25E-01 |
| rs12621220 | IL-1B | t | c | 0.277 | 1.080 | (0.979-1.192) | 1.26E-01 |
| rs1143627 | IL-1B | a | g | 0.656 | 0.937 | (0.853-1.029) | 1.74E-01 |
| rs1143634 | IL-1B | a | g | 0.244 | 0.952 | (0.856-1.058) | 3.61E-01 |
| rs1143643 | IL-1B | t | c | 0.336 | 0.987 | (0.897-1.086) | 7.91E-01 |
| rs1143633 | IL-1B | t | c | 0.337 | 0.988 | (0.898-1.087) | 8.03E-01 |
| rs3917368 | IL-1B | t | c | 0.335 | 0.990 | (0.901-1.089) | 8.42E-01 |
| | | | | | | | |
| rs1800469 | TGFb | a | g | 0.299 | 0.931 | (0.843-1.029) | 1.61E-01 |
| | | | | | | | |
| rs11661134 | MC2R | a | g | 0.069 | 0.859 | (0.692-1.067) | 1.70E-01 |
| | | | | | | | |
| rs11627241 | SERPINA6 | t | c | 0.257 | 0.931 | (0.839-1.033) | 1.78E-01 |
| rs746530 | SERPINA6 | a | g | 0.327 | 0.940 | (0.854-1.034) | 2.01E-01 |
| rs941601 | SERPINA6 | t | C | 0.135 | 0.954 | (0.837-1.087) | 4.76E-01 |
| rs1998056 | SERPINA6 | c | g | 0.413 | 1.019 | (0.931-1.115) | 6.86E-01 |
| rs8022616 | SERPINA6 | a | g | 0.892 | 1.005 | (0.867-1.165) | 9.48E-01 |
| | | | | | | | |
| rs1800871 | IL-10 | a | g | 0.220 | 0.931 | (0.834-1.038) | 1.99E-01 |
| rs1800872 | IL-10 | t | g | 0.221 | 0.931 | (0.834-1.039) | 2.02E-01 |
| rs1800896 | IL-10 | t | c | 0.492 | 0.968 | (0.886-1.057) | 4.73E-01 |
| rs1800890 | IL-10 | a | t | 0.600 | 1.001 | (0.910-1.103) | 9.77E-01 |
| | | | | | | | |
| rs1875999 | CRHBP | t | c | 0.683 | 0.948 | (0.863-1.041) | 2.61E-01 |
| | | | | | | | |
| rs17561 | IL-1A | a | c | 0.304 | 0.950 | (0.862-1.047) | 3.02E-01 |
| rs1800587 | IL-1A | a | g | 0.304 | 0.951 | (0.862-1.048) | 3.08E-01 |

| MarkerName (III) | Gene | Allele1 | Allele2 | Freq A1 | OR | 95%CI | P-value |
|---|---|---|---|---|---|---|---|
| rs3813034 | SLC6A4 | a | c | 0.538 | 1.044 | (0.955-1.141) | 3.46E-01 |
| | | | | | | | |
| rs11842874 | MCF2L | a | g | 0.931 | 1.098 | (0.903-1.335) | 3.51E-01 |
| | | | | | | | |
| rs2239704 | TNF / LTA | a | c | 0.387 | 0.957 | (0.872-1.050) | 3.54E-01 |
| | | | | | | | |
| rs6746030 | SCN9A | a | g | 0.140 | 1.058 | (0.932-1.202) | 3.81E-01 |
| | | | | | | | |
| rs454078 | IL-1RN | a | t | 0.730 | 0.964 | (0.874-1.064) | 4.72E-01 |
| rs315952 | IL-1RN | T | c | 0.705 | 0.990 | (0.899-1.090) | 8.31E-01 |
| | | | | | | | |
| rs1048101 | ADRA1A | a | g | 0.562 | 0.971 | (0.889-1.061) | 5.19E-01 |
| | | | | | | | |
| rs1946518 | IL-18 | t | g | 0.397 | 1.028 | (0.940-1.125) | 5.38E-01 |
| | | | | | | | |
| rs1799945 | HFE | c | g | 0.858 | 1.035 | (0.910-1.177) | 6.01E-01 |
| | | | | | | | |
| rs2842003 | RGS4 | t | g | 0.428 | 1.021 | (0.929-1.123) | 6.65E-01 |
| | | | | | | | |
| rs6280 | DRD3 | t | c | 0.676 | 1.021 | (0.929-1.123) | 6.69E-01 |
| | | | | | | | |
| rs1020759 | NFKb | t | c | 0.402 | 0.980 | (0.896-1.073) | 6.69E-01 |
| | | | | | | | |
| rs4129256 | TAAR2 | a | g | 0.203 | 1.024 | (0.917-1.143) | 6.72E-01 |
| rs2745428 | TAAR2 | a | c | 0.788 | 0.993 | (0.892-1.106) | 9.03E-01 |
| | | | | | | | |
| rs5275 | PTGS2 | a | g | 0.671 | 1.020 | (0.929-1.121) | 6.75E-01 |
| | | | | | | | |
| rs4906902 | GABRB3 | a | g | 0.827 | 1.026 | (0.910-1.156) | 6.80E-01 |
| | | | | | | | |
| rs2069827 | IL-6 | t | g | 0.058 | 0.960 | (0.781-1.179) | 6.96E-01 |
| rs1800795 | IL-6 | c | g | 0.409 | 1.004 | (0.916-1.099) | 9.39E-01 |
| rs1800797 | IL-6 | a | g | 0.399 | 0.997 | (0.909-1.093) | 9.44E-01 |
| rs1554606 | IL-6 | t | g | 0.428 | 0.998 | (0.913-1.091) | 9.72E-01 |
| rs2069845 | IL-6 | a | g | 0.572 | 1.001 | (0.916-1.095) | 9.76E-01 |
| | | | | | | | |
| rs7911 | GBP1 | a | g | 0.637 | 0.994 | (0.907-1.090) | 9.05E-01 |
| | | | | | | | |
| rs6265 | BDNF | t | c | 0.198 | 1.006 | (0.900-1.124) | 9.22E-01 |

**ACKNOWLEDGMENTS**

This study was funded by the European Commission (HEALTH-F2-2008-201865, GEFOS; HEALTH-F2-2008 35627, TREAT-OA), the Netherlands Organisation for Scientific Research (NWO) Investments (nr. 175.010.2005.011, 911-03-012), the Netherlands Consortium for Healthy Aging (NCHA), the Netherlands Genomics Initiative (NGI) / Netherlands Organisation for Scientific Research (NWO) project nr. 050-060-810 and VIDI grant 917103521, and a Rubicon fellowship; Research Institute for Diseases in the Elderly (RIDE) grant 94800012; The Wellcome Trust and Arthritis Research UK.

**1958BC (British 1958 birth cohort)** – Phenotype data and sample collection for the Biomedical sweep was supported by the Medical Research Council, UK (Health of the Public initiative grant G0000934). Genotype data was provided by the Wellcome Trust Case-Control Consortium (WTCCC) and the Type 1 Diabetes Genetics Consortium (T1DGC). A full list of the investigators who contributed to generation of the WTCCC data is available from www.wtccc.org.uk; funding was provided by the Wellcome Trust under award 076113. T1DGC is collaborative clinical study sponsored by the National Institute of Diabetes and Digestive and Kidney Diseases (NIDDK), National Institute of Allergy and Infectious Diseases (NIAID), National Human Genome Research Institute (NHGRI), National Institute of Child Health and Human Development (NICHD), and Juvenile Diabetes Research Foundation International (JDRF) and supported by U01 DK062418.

**AGES (Age, Gene/Environment Susceptibility-Reykjavik Study)** - The AGES Reykjavik Study has been funded by NIH contracts N01-AG-12100 and Z01-AG-007380, the NIA Intramural Research Program, Hjartavernd (the Icelandic Heart Association), and the Althingi (the Icelandic Parliament). The study is approved by the Icelandic National Bioethics Committee,

(VSN: 00-063) and the Data Protection Authority. The researchers are indebted to the participants for their willingness to participate in the study.

**CHINGFORD (The Chingford Study) -** We would like to thank all the participants of the Chingford Women Study and Maxine Daniels and Dr Alan Hakim for their time and dedication and Arthritis Research UK for their funding support to the study and the Oxford NIHR Musculoskeletal Biomedical Research Unit for funding contributions.

**DSDBAC (The Dyne Steel DNA Bank for Ageing and Cognition) –** Genotyping of this cohort was supported by the BBSRC and the collection of phenotype data used in this study was supported by AgeUK and Arthritis Research UK. Ethical approval was obtained from the University of Manchester.

**EPIFUND (The EPIdemiological study of FUNctional Disorderes) –** The EPIFUND study was supported by Arthritis Research UK (17552) and was conducted with the approval of the local research ethics committee and the University of Manchester. The researchers would like to thank the participants and staff at the three general practices who contributed to the study and administrative and laboratory staff at the Arthritis Research UK Epidemiology Unit for their contributions to the project.

**ERF (Erasmus Rucphen Family) (EUROSPAN) -** The genotyping for the ERF study was supported by EUROSPAN (European Special Populations Research Network) and the European Commission FP6 STRP grant (018947; LSHG-CT-2006-01947). The ERF study was further supported by grants from the Netherlands Organisation for Scientific Research, Erasmus MC, the Centre for Medical Systems Biology (CMSB) and the Netherlands Brain Foundation (HersenStichting Nederland). We are grateful to all patients and their relatives, general practitioners and neurologists for their contributions and to Petra Veraart for her help in genealogy, Jeannette Vergeer for the supervision of the laboratory work and Peter Snijders for his help in data collection.

**FOA (The Framingham Osteoarthritis Study) -** The study was supported by grants from the National Heart, Lung, and Blood Institute (NHLBI contract N01-HC-25195) and NIH AR47785, AG18393, and AR550127. The researchers thank the study participants and personnel.

**GARP (The Genetics osteoarthritis and Progression Study) -** This study was supported the Leiden University Medical Centre and the Dutch Arthritis Association. Pfizer Inc., Groton, CT, USA supported the inclusion of the GARP study. The genotypic work was supported by the Netherlands Organization of Scientific Research (MW 904-61-095, 911-03-016, 917 66344 and 911-03-012), Leiden University Medical Centre and the Centre of Medical System Biology and Netherlands Consortium for Healthy Aging both in the framework of the Netherlands Genomics Initiative (NGI). The research leading to these results has received funding from the European Union's Seventh Framework Programme (FP7/2007-2011) under grant agreement n° 259679.

**HCS (The Hertfordshire Cohort Study) –** This study was funded by the Medical Research Council of Great Britain, Arthritis Research UK, the International Osteoporosis Foundation and the NIHR Nutrition BRU, University of Southampton.

**RS (The Rotterdam Study)** - The GWA study was funded by the Netherlands Organisation of Scientific Research NWO Investments (nr. 175.010.2005.011, 911-03-012), the Research Institute for Diseases in the Elderly (014-93-015; RIDE2), the Netherlands Genomics Initiative (NGI)/Netherlands Consortium for Healthy Aging (NCHA) project nr. 050-060-810. We thank Pascal Arp, Mila Jhamai, Dr Michael Moorhouse, Marijn Verkerk, and Sander Bervoets for their help in creating the GWAS database. The Rotterdam Study is funded by Erasmus Medical Center and Erasmus University, Rotterdam, Netherlands Organization for the Health Research and Development (ZonMw), the Research Institute for Diseases in the Elderly (RIDE), the Ministry of Education, Culture and Science, the Ministry for Health, Welfare and Sports, the European Commission (DG XII), and the Municipality of Rotterdam. The authors are very grateful to the participants and staff from the Rotterdam Study, the participating general practitioners and the

pharmacists. We would like to thank Dr. Tobias A. Knoch, Luc V. de Zeeuw, Anis Abuseiris, and Rob de Graaf as well as their institutions the Erasmus Computing Grid, Rotterdam, The Netherlands, and especially the national German MediGRID and Services@MediGRID part of the German D-Grid, both funded by the German Bundesministerium fuer Forschung und Technology under grants #01 AK 803 A-H and # 01 IG 07015 G for access to their grid resources.

**SHIP (The Study of Health in Pomerania)** - SHIP is part of the Community Medicine Research net of the University of Greifswald, Germany, which is funded by the Federal Ministry of Education and Research (grants no. 01ZZ9603, 01ZZ0103, and 01ZZ0403), the Ministry of Cultural Affairs as well as the Social Ministry of the Federal State of Mecklenburg-West Pomerania. Genome-wide data have been supported by the Federal Ministry of Education and Research (grant no. 03ZIK012) and a joint grant from Siemens Healthcare, Erlangen, Germany and the Federal State of Mecklenburg- West Pomerania. The University of Greifswald is a member of the ‘Center of Knowledge Interchange’ program of the Siemens AG. Data analyses were further supported by the DIAB Core project of the German Network of Diabetes.

**TwinsUK (The UK Adult Twin Registry) -** This study was funded by the Wellcome Trust (Grant ref. 079771); European Community’s Seventh Framework Programme (FP7/2007-2013)/grant agreement HEALTH-F2-2008-ENGAGE and the European Union FP-5 GenomEUtwin Project (QLG2-CT-2002-01254) and Framework 6 Project EUroClot. The study also receives support from the National Institute for Health Research (NIHR) comprehensive Biomedical Research Centre award to Guy's & St Thomas' NHS Foundation Trust in partnership with King's College London. We thank the staff from the TwinsUK, the DNA Collections and Genotyping Facilities at the Wellcome Trust Sanger Institute for sample preparation; Quality Control of the Twins UK cohort for genotyping (in particular Amy Chaney, Radhi Ravindrarajah, Douglas Simpkin, Cliff Hinds, and Thomas Dibling); Paul Martin and Simon Potter of the DNA and Genotyping Informatics teams for data handling; Le Centre National de Génotypage, France,

led by Mark Lathrop, for genotyping; Duke University, North Carolina, USA, led by David Goldstein, for genotyping; and the Finnish Institute of Molecular Medicine, Finnish Genome Center, University of Helsinki, led by Aarno Palotie. Nicole Soranzo acknowledges financial support from the Wellcome Trust (Grant 091746/Z/10/Z).

**University Medical Center Utrecht -** The work of Annemieke Kavelaars is supported by NIH grants RO1 NS074999 and RO1 NS073939.

**The authors are very grateful to all study participants, the staff from all studies and the participating physicians and pharmacists.**

**References:**

1. Teo YY, Inouye M, Small KS*, et al.* A genotype calling algorithm for the Illumina BeadArray platform. Bioinformatics. 2007 Oct 15;23(20):2741-6.

2. Li Y, Willer CJ, Ding J*, et al.* MaCH: using sequence and genotype data to estimate haplotypes and unobserved genotypes. Genet Epidemiol. 2010 Dec;34(8):816-34.

3. Zhao Z, Timofeev N, Hartley SW*, et al.* Imputation of missing genotypes: an empirical evaluation of IMPUTE. BMC Genet. 2008;9:85.

4. Biostat. Comprehensive Meta-Analysis Software. 2011 [cited 2011-03-04]; Available from: http://www.meta-analysis.com/.

5. Pruim RJ, Welch RP, Sanna S*, et al.* LocusZoom: regional visualization of genome-wide association scan results. Bioinformatics. 2010 Sep 15;26(18):2336-7.

6. Aulchenko YS, Heutink P, Mackay I*, et al.* Linkage disequilibrium in young genetically isolated Dutch population. Eur J Hum Genet. 2004 Jul;12(7):527-34.

7. Hofman A, van Duijn CM, Franco OH*, et al.* The Rotterdam Study: 2012 objectives and design update. Eur J Epidemiol. 2011 Aug;26(8):657-86.

8. Spector TD, Williams FM. The UK Adult Twin Registry (TwinsUK). Twin Res Hum Genet. 2006 Dec;9(6):899-906.

9. Spector TD, MacGregor AJ. The St. Thomas' UK Adult Twin Registry. Twin Res. 2002 Oct;5(5):440-3.

10. Power C, Atherton K, Manor O. Co-occurrence of risk factors for cardiovascular disease by social class: 1958 British birth cohort. J Epidemiol Community Health. 2008 Dec;62(12):1030-5.

11. Hocking LJ, Smith BH, Jones GT*, et al.* Genetic variation in the beta2-adrenergic receptor but not catecholamine-O-methyltransferase predisposes to chronic pain: results from the 1958 British Birth Cohort Study. Pain. 2010 Apr;149(1):143-51.

12. Harris TB, Launer LJ, Eiriksdottir G*, et al.* Age, Gene/Environment Susceptibility-Reykjavik Study: multidisciplinary applied phenomics. Am J Epidemiol. 2007 May 1;165(9):1076-87.

13. Rabbitt P, McInnes L, Diggle P*, et al.* The University of Manchester longitudinal study of cognition in normal healthy old age, 1983 through 2003. Aging, Neuropsychology and Cognition. 2004;11:P245-79.

14. Felson DT, Zhang Y, Hannan MT, *et al.* The incidence and natural history of knee osteoarthritis in the elderly. The Framingham Osteoarthritis Study. Arthritis Rheum. 1995 Oct;38(10):1500-5.

15. Riyazi N, Meulenbelt I, Kroon HM, *et al.* Evidence for familial aggregation of hand, hip, and spine but not knee osteoarthritis in siblings with multiple joint involvement: the GARP study. Ann Rheum Dis. 2005 Mar;64(3):438-43.

16. John U, Greiner B, Hensel E, *et al.* Study of Health In Pomerania (SHIP): a health examination survey in an east German region: objectives and design. Soz Praventivmed. 2001;46(3):186-94.

17. Volzke H, Alte D, Schmidt CO, *et al.* Cohort profile: the study of health in Pomerania. Int J Epidemiol. 2011 Apr;40(2):294-307.

18. Hart DJ, Spector TD. The relationship of obesity, fat distribution and osteoarthritis in women in the general population: the Chingford Study. J Rheumatol. 1993 Feb;20(2):331-5.

19. Hart DJ, Spector TD. Cigarette smoking and risk of osteoarthritis in women in the general population: the Chingford study. Ann Rheum Dis. 1993 Feb;52(2):93-6.

20. Holliday KL, Nicholl BI, Macfarlane GJ, *et al.* Genetic variation in the hypothalamic-pituitary-adrenal stress axis influences susceptibility to musculoskeletal pain: results from the EPIFUND study. Ann Rheum Dis. 2010 Mar;69(3):556-60.

21. Syddall HE, Aihie Sayer A, Dennison EM, *et al.* Cohort profile: the Hertfordshire cohort study. Int J Epidemiol. 2005 Dec;34(6):1234-42.

22. Aulchenko YS, Ripke S, Isaacs A, *et al.* GenABEL: an R library for genome-wide association analysis. Bioinformatics. 2007 May 15;23(10):1294-6.

23. Estrada K, Abuseiris A, Grosveld FG, *et al.* GRIMP: a web- and grid-based tool for high-speed analysis of large-scale genome-wide association using imputed data. Bioinformatics. 2009 Oct 15;25(20):2750-2.

24. Purcell S, Neale B, Todd-Brown K, *et al.* PLINK: a tool set for whole-genome association and population-based linkage analyses. Am J Hum Genet. 2007 Sep;81(3):559-75.
